# A Novel Approach to Clustering Genome Sequences Using Inter-nucleotide Covariance

**DOI:** 10.3389/fgene.2019.00234

**Published:** 2019-04-09

**Authors:** Rui Dong, Lily He, Rong Lucy He, Stephen S.-T. Yau

**Affiliations:** ^1^Department of Mathematical Sciences, Tsinghua University, Beijing, China; ^2^Department of Biological Sciences, Chicago State University, Chicago, IL, United States

**Keywords:** accumulated natural vector, phylogenetic analysis, alignment-free, inter-nucleotide covariance, genomes

## Abstract

Classification of DNA sequences is an important issue in the bioinformatics study, yet most existing methods for phylogenetic analysis including Multiple Sequence Alignment (MSA) are time-consuming and computationally expensive. The alignment-free methods are popular nowadays, whereas the manual intervention in those methods usually decreases the accuracy. Also, the interactions among nucleotides are neglected in most methods. Here we propose a new Accumulated Natural Vector (ANV) method which represents each DNA sequence by a point in ℝ^18^. By calculating the Accumulated Indicator Functions of nucleotides, we can further find an Accumulated Natural Vector for each sequence. This new Accumulated Natural Vector not only can capture the distribution of each nucleotide, but also provide the covariance among nucleotides. Thus global comparison of DNA sequences or genomes can be done easily in ℝ^18^. The tests of ANV of datasets of different sizes and types have proved the accuracy and time-efficiency of the new proposed ANV method.

## Introduction

With the rapid development of Next Generation Sequencing technology, more and more information of the genome sequences is available. Studying sequence similarity is a crucial question in research and can explain phylogenetic relationships by constructing trees. One of the most commonly used methods, Multiple Sequence Alignment (MSA) uses dynamic programming, a regression technique that finds an optimal alignment by assigning scores to different possible alignments and taking the one with the highest score (Yu et al., 2013a). However, the computational cost of MSA is extremely high and MSA may not produce accurate phylogeny for diverse systems of different families of RNA viruses (Yu et al., 2013b). Alignment-free approaches have been developed to overcome those limitations. Published alignment-free methods include Markov chain models (Apostolico and Denas, 2008), chaos theory (Hatje and Kollmar, 2012), and some other methods based on the statistics of oligomer frequency and associated with a fixed length segment, known as *k-mer* (Sims et al., 2009). Yau and his team proposed the natural vector method, which takes the position of each nucleotide into consideration. The natural vector method performs well on many datasets (Deng et al., 2011; Yu et al., 2013b; Hoang et al., 2016; Li et al., 2016), however, it only considers the number, average position and dispersion of positions of each nucleotide. Relationships between nucleotides are also important, especially when the functions may be related to interactions of nucleotides, such as the folding of a chromosome. In this paper, we propose a new Accumulated Natural Vector (ANV) method, which not only considers the basic property of each nucleotide, but also the covariance between them. In the traditional Natural Vector (NV) method, each sequence is uniquely represented by a single point in ℝ^12^. The traditional Natural Vector approach is firstly introduced in Deng et al. (2011): for a sequence of length N, *n*_α_ (αϵ{*A, C, T, G*}) denotes the number of nucleotide α in the sequence. s[α][v] is the distance from the first nucleotide (regarded as origin) to the *v*^*th*^ nucleotide α in the DNA sequence. $T_{\alpha }=\sum_{v=1}^{n_{\alpha }}\text{s}\left[\alpha \right]\left[\text{v}\right]$ denotes the total distance of each set of A,C,G,T from the origin, αϵ{*A, C, T, G*}. $\mu_{\alpha }=\frac{T_{\alpha }}{n_{\alpha }}$, is the mean value of the distances of nucleotide α from the origin. $D_{2}^{\alpha }=\sum_{v=1}^{n_{\alpha }}\frac{{\left(\text{s}\left[\alpha \right]\left[\text{v}\right]-\mu_{\alpha }\right)}^{2}}{n_{\alpha }\times N}$, is the normalized central moment of order 2, which can also be seen as the variance of the positions of nucleotide α. Therefore, a DNA sequence can be represented by a 12-dim vector:

$$\begin{array}{l} \left(n_{A},n_{C},n_{G},n_{T},\mu_{A},\mu_{c},\mu_{G},\mu_{T},D_{2}^{A},D_{2}^{C},D_{2}^{G},D_{2}^{T}\right) \end{array}$$

In this paper, we propose an Accumulated Natural Vector approach, which projects each sequence into a point in ℝ^18^, where the additional six dimensions describe the covariance between nucleotides. Obviously, ANV can provide more information than the traditional NV method, and doesn't include the human intervention, such as choosing the optimal value of k in the *k-mer* method. Therefore, it can distinguish different sequences and classify species into correct clusters with higher accuracy and less time cost.

## Materials and Methods

### Materials

The following six datasets were used to validate the method. The Coronaviruses dataset includes 36 viral genomes, in which 34 viruses are from the exact same dataset with (Woo et al., 2005; Yu et al., 2010; Hoang et al., 2015) and the other two viruses are new members in Coronavirus. The second dataset consists of the genomes of 38 Influenza A viruses, which is a classic dataset to test if a new proposed method performs well. The third dataset includes 72 viruses from Zheng et al. (2015), which focuses on the classification of Ebolaviruses. The fourth one is from our colleagues' previous paper (Li et al., 2016) which includes 351 viruses chosen randomly under some criteria. The fifth one is the mitochondrial genomes of 31 mammals, which can be clustered into seven well-known categories. All the sequence materials can be found on NCBI with the reference number provided in the Appendices. We also generated different mutations by simulation in a DNA sequence and constructed phylogenetic trees of simulated sequences to test our ANV method.

All computations in this paper are done on a Dell laptop equipped with Intel i7 Processor under Windows 10 Home Premium with 8 GB RAM, together with the Matlab (version R2017a) and Mega X.

### Methods

#### Indicator Function

For a given genomic sequence, we first define four Indicator Functions (u) for Adenine, Cytosine, Guanine and Thymine, respectively:

(1) $$u_{\alpha }\left(i\right)=\{{}_{\text{​​​​​​​​​​​}0,\;\;\;\;\;if\;\alpha \;doesn\prime t\;appear\;at\;the\;i^{th}\;position\;of\;the\;sequence}^{1,\;\;\;\;\;\;\;\;\;\;if\;\alpha \;appears\;at\;the\;i^{th}\;position\;of\;the\;sequence}$$

where αϵ{*A, C, T, G*}, and i = 1, 2, …, N. HereN is the length of the whole sequence.

For example, if the genomic sequence is “*ATCTAGCT*,” then the four Indicator Functions are shown in Table 1.

**Table 1:** The Indicator Functions of the sequence “ATCTAGCT”.

**Sequence**	**A**	**T**	**C**	**T**	**A**	**G**	**C**	**T**
Position(i)	1	2	3	4	5	6	7	8
*u*_*A*_ (i)	1	0	0	0	1	0	0	0
*u*_*C*_ (i)	0	0	1	0	0	0	1	0
*u*_*G*_ (i)	0	0	0	0	0	1	0	0
*u*_*T*_ (i)	0	1	0	1	0	0	0	1

Here are some simple properties about the Indicator Functions:

1. Each column has the sum of 1.
  (2) $$\begin{array}{l} \sum \underset{\alpha \in \left\{A,C,G,T\right\}}{}u_{\alpha }\left(i\right)=1,for\text{ i}=1,2,\ldots ,\text{N} \end{array}$$
2. Each row has the sum of the number of corresponding nucleotide.
  (3) $$\begin{array}{l} n_{\alpha }=\sum_{i=1}^{N}u_{\alpha }\left(i\right),\alpha \in \left\{A,C,G,T\right\} \end{array}$$

#### Accumulated Indicator Function

Now we define four Accumulated Indicator Functions as the following:

(4) $$\begin{array}{l} {ũ}_{\alpha }\left(N\right)=\sum_{i=1}^{N}u_{\alpha }\left(i\right) \end{array}$$

The four Accumulated Indicator Functions for the example above (“*ATCTAGCT”*), are shown in Table 2.

**Table 2:** The Accumulated Indicator Functions of the sequence “ATCTAGCT.”

**Sequence**	**A**	**T**	**C**	**T**	**A**	**G**	**C**	**T**
Position(i)	1	2	3	4	5	6	7	8
*ũ*_*A*_(*i*)	1	1	1	1	2	2	2	2
*ũ*_*C*_(*i*)	0	0	1	1	1	1	2	2
*ũ*_*G*_(*i*)	0	0	0	0	0	1	1	1
*ũ*_*T*_(*i*)	0	1	1	2	2	2	2	3

Here are some properties about the Accumulated Indicator Functions:

The *i*^*th*^ column has the sum of i.
  (5) $$\begin{array}{l} \sum_{\alpha \in \left\{A,C,G,T\right\}}{ũ}_{\alpha }\left(i\right)=i \end{array}$$
The last column is the total number of the nucleotide α in the sequence.
  (6) $$\begin{array}{l} {ũ}_{\alpha }\left(N\right)=n_{\alpha } \end{array}$$
  (7) $$\overset{N}{\underset{i=1}{\sum }}{ũ}_{\alpha }\left(i\right)=n_{\alpha }\times \left(N+1-\mu_{\alpha }\right)$$

μ_*k*_ is the average position in the Natural Vector in Deng et al. (2011).

Property 1 and 2 can be easily proved by the definition of Indicator Function (*u*_α_) and Accumulated Indicator Function (*ũ*_α_), now we prove the Property 3, which builds up the relationship between the Accumulated Indicator Function and the average position of a specific nucleotide.

If we assume that the positions of nucleotide α are *t*_1_, *t*_2_, …, *t*_*n*_α__, where *n*_α_ is the number of nucleotide α in the sequence, then the basic form of Accumulated Indicator Function should be, which satisfies 1 ≤ *t*_1_ < *t*_2_ < … < *t*_*i*−1_ < *t*_*i*_ < … < *t*_*n*_α__ ≤ *N*,

$$\begin{array}{l} {}[\underset{t_{1}\;-\;1}{\underset{︸}{0,0,\;\ldots \;,\;0,}}\underset{\left(t_{2}\;-\;1\right)\;-\;\left(t_{1}\;-\;1\right)\;}{\underset{︸}{1,1,\;\ldots \;,1,}}2,2,\;\ldots \;,2,\;\ldots \\ \underset{\left(t_{n_{\alpha }}\;-\;1\right)\;-\;\left(t_{n_{\alpha }-1}\;-\;1\right)}{\underset{︸}{\left(n_{\alpha }-1\right),\left(n_{\alpha }-1\right),\;\ldots \left(n_{\alpha }-1\right)}},\underset{N\;-\;t_{n_{\alpha }}\;+\;1}{\underset{︸}{n_{\alpha },\;\ldots ,n_{\alpha }]}} \end{array}$$

If we add up those N elements above and denote the sum as Ω_α_ and *t*_0_ = 0, we have:

(8) $$\begin{array}{l} \Omega_{\alpha }=\overset{n_{\alpha }}{\underset{i=1}{\sum }}\left(t_{i}-t_{i-1}\right)\times \left(i-1\right)+\left(N-t_{n_{\alpha }}+1\right)\times n_{\alpha }\\ =\left(t_{2}-t_{1}\right)\times 1+\left(t_{3}-t_{2}\right)\times 2+\left(t_{4}-t_{3}\right)\times 3+\ldots \\ +\left(t_{n_{\alpha }}-t_{n_{\alpha }-1}\right)\times \left(n_{\alpha }-1\right)+\left(N-t_{n_{\alpha }}+1\right)\times n_{\alpha }\\ =-t_{1}-t_{2}-t_{3}-\ldots -t_{n_{\alpha }-1}+\left(n_{\alpha }-1\right)\times t_{n_{\alpha }}+\ldots \\ +\left(N-t_{n_{\alpha }}+1\right)\times n_{\alpha }\\ =-t_{1}-t_{2}-t_{3}-\ldots -t_{n_{\alpha }-1}-t_{n_{\alpha }}+\left(N+1\right)\times n_{\alpha }\\ =-n_{\alpha }\times \mu_{\alpha }+\left(N+1\right)\times n_{\alpha } \end{array}$$

Thus, we have

(9) $$\begin{array}{l} \sum_{i=1}^{N}{ũ}_{\alpha }\left(i\right)=n_{\alpha }\times \left(N+1-\mu_{\alpha }\right) \end{array}$$

Therefore, we use

(10) $$\begin{array}{l} \zeta_{\alpha }=\frac{\Omega_{\alpha }}{n_{\alpha }} \end{array}$$

to describe the average position of nucleotide α, which indicates the distance of the average position to the end of the sequence.

#### Inter-nucleotide Covariance

For two finite point sets with equal number of elements: A = {*a*_1_, *a*_2_, …, *a*_*n*_}, B = {*b*_1_, *b*_2_, …, *b*_*n*_} in ℝ, which satisfy *a*_1_ < *a*_2_ < … < *a*_*n*_ and *b*_1_ < *b*_2_ < … < *b*_*n*_, the covariance of two sets can be defined as follows:

(11) $$\begin{array}{l} \text{cov}\left(\text{A},\text{B}\right)=\sum_{i=1}^{n}\frac{\left(a_{i}-u_{A}\right)\times \left(b_{i}-u_{B}\right)}{a_{n}\times b_{n}} \end{array}$$

where $u_{A}=\sum_{i=1}^{n}a_{i}/n$ and $u_{B}=\sum_{i=1}^{n}b_{i}/n$.

Now we apply the covariance formula above to the Accumulated Indicator Functions. A set is a collection of definite, distinct objects, known as the elements or members of the set. Now for each nucleotide, we have an array of N elements which is the Accumulated Indicator Function for the nucleotide α ∈ {*A, C, G, T*}:

$$\begin{array}{l} {}[0,0,\ldots ,0,1,1,\ldots ,1,2,2,\ldots ,2,\ldots \left(n_{\alpha }-1\right),\\ \;(n_{\alpha }-1),\ldots ,(n_{\alpha }-1),n_{\alpha },\ldots ,n_{\alpha }] \end{array}$$

However, those N elements cannot build up a set of N elements since many of them are replicated. Hence, we extend the definition of set to a generalized concept, where the elements in a set can be the same. In this generalized definition, each nucleotide has a set of N elements and they can be arranged in the ascending order, i.e., from the smallest to the biggest number. Thus, we can use the covariance formula (11). As the example of sequence “*ATCTAGCT*,” the covariance of nucleotide A and C can be computed in this way: the generalized set of nucleotide A is {1,1,1,1,2,2,2,2} and of C is {0,0,1,1,1,1,2,2}. Each generalized set has N = 8 elements and the generalized covariance would be

(12) $$\begin{array}{l} \theta_{A}=\sum_{i=1}^{N}{ũ}_{A}\left(i\right)/N=\frac{1+1+1+1+2+2+2+2}{8}=1.5 \end{array}$$

(13) $$\begin{array}{l} \theta_{C}=\sum_{i=1}^{N}{ũ}_{C}\left(i\right)/N=\frac{0+0+1+1+1+1+2+2}{8}=1 \end{array}$$

(14) $$\begin{array}{l} \text{ cov}\left(\text{A},\text{C}\right)=\underset{i\;=\;1}{\sum^{N}}\frac{\left({\tilde{u}}_{A}\left(i\right)-\theta_{A}\right)\times \left({\tilde{u}}_{C}\left(i\right)-\theta_{C}\right)}{n_{A}\times n_{C}}\\ =\frac{1}{2\times 2}\times [\left(1-1.5\right)\times \left(0-1\right)+\left(1-1.5\right)\times \left(0-1\right)\\ \;+\left(1-1.5\right)\times \left(1-1\right)+\left(1-1.5\right)\times \left(1-1\right)\\ \;+\left(2-1.5\right)\times \left(1-1\right)+\left(2-1.5\right)\times \left(1-1\right)\\ \;+\left(2-1.5\right)\times \left(2-1\right)+\left(2-1.5\right)\times \left(2-1\right)]\\ \;=\frac{1}{2} \end{array}$$

Similarly, we can get cov(A, G), cov(A, T), cov(C, G), cov(C, T), cov(G, T).

#### Compatibility of Variance and Covariance

For two nucleotides like α and β, the covariance formula is

(15) $$\begin{array}{l} \text{cov}\left(\alpha ,\beta \right)=\sum_{i=1}^{N}\frac{\left({ũ}_{\alpha }\left(i\right)-\theta_{\alpha }\right)\times \left({ũ}_{\beta }\left(i\right)-\theta_{\beta }\right)}{n_{\alpha }\times n_{\beta }} \end{array}$$

Then it is obvious that when α = β, the corresponding formula should be

(16) $$\begin{array}{l} D_{\alpha }=\text{cov}\left(\alpha ,\alpha \right)=\sum_{i=1}^{N}\frac{\left({ũ}_{\alpha }\left(i\right)-\theta_{\alpha }\right)\times \left({ũ}_{\alpha }\left(i\right)-\theta_{\alpha }\right)}{n_{\alpha }\times n_{\alpha }}\\ =\sum_{i=1}^{N}{\left(\frac{{ũ}_{\alpha }\left(i\right)-\theta_{\alpha }}{n_{\alpha }}\right)}^{2} \end{array}$$

The formula above defines the variance of the positions of nucleotide α.

#### Accumulated Natural Vector

For a given nucleotide sequence, now we can build up its Accumulated Natural Vector. The first four dimensions describe the number of each nucleotide, denoted as *n*_*A*_, *n*_*C*_, *n*_*G*_, *n*_*T*_, which are the last column of the Accumulated Indicator Functions. The second four dimensions describe the average distance of nucleotides to the end of the sequence, denoted as $\zeta_{A}=\frac{\Omega_{A}}{n_{A}},\zeta_{c}=\frac{\Omega_{C}}{n_{C}},\zeta_{G}=\frac{\Omega_{G}}{n_{G}},\zeta_{T}=\frac{\Omega_{T}}{n_{T}}$ as formula (10). The third four dimensions describe the divergence of each nucleotide, denoted as $D_{A}=\sum_{i=1}^{N}{\left(\frac{{ũ}_{A}\left(i\right)-\theta_{A}}{n_{A}}\right)}^{2},D_{C}=\sum_{i=1}^{N}{\left(\frac{{ũ}_{C}\left(i\right)-\theta_{C}}{n_{C}}\right)}^{2},D_{G}=\sum_{i=1}^{N}{\left(\frac{{ũ}_{G}\left(i\right)-\theta_{G}}{n_{G}}\right)}^{2},D_{T}=\sum_{i=1}^{N}{\left(\frac{{ũ}_{T}\left(i\right)-\theta_{T}}{n_{T}}\right)}^{2}$as formula (16). Please note that this *D*_α_ is a little different from the $D_{2}^{\alpha }$ in the traditional Natural Vector method since the previous definition of variance cannot be extended to a reliable definition of covariance. The last six dimensions describe the covariances between each two nucleotides, denoted as cov(A, G), cov(A, T), cov(C, G), cov(C, T), cov(G, T) as formula (15). And the universal form of Accumulated Natural Vector is

$$\begin{array}{l} \;(n_{A},n_{C},n_{G},n_{T},\zeta_{A},\zeta_{c},\zeta_{G},\zeta_{T},D_{A},D_{C},D_{G},D_{T},\\ \text{cov}\left(\text{A},\text{C}\right)\text{​},\text{cov}\left(\text{A},\text{G}\right)\text{​},\text{cov}\left(\text{A},\text{T}\right)\text{​},\text{cov}\left(\text{C},\text{G}\right)\text{​},\text{cov}\left(\text{C},\text{T}\right)\text{​},\text{cov}\left(\text{G},\text{T}\right)) \end{array}$$

#### Euclidean Distances Between Accumulated Natural Vectors

From section 2.2.1 to section 2.2.5, we introduce how a DNA sequence is represented by a vector in ℝ^18^ space. Therefore, the distance between two sequences can be measured by the Euclidean distance between two vectors. Suppose that now we have two sequences in ℝ^*w*^ (in our case, w = 18), denoted as x = (*x*_1_, …, *x*_*w*_) and (*y*_1_, …, *y*_*w*_), the Euclidean distance between them is

(17) $$\begin{array}{l} \text{d}\left(\text{x},\text{y}\right)={\left(\sum_{i=1}^{w}{\left(x_{i}-y_{i}\right)}^{2}\right)}^{\frac{1}{2}} \end{array}$$

For a dataset of m different sequences, we can construct a distance matrix D = (*d*_*ij*_)_*m*×*m*_, and *d*_*ij*_(≥ 0) represents the Euclidean distance between sequence i and sequence j. D is a symmetric matrix and the diagonal element is zero.

#### Constructing Phylogenetic Trees and Comparisons

In this research, we use Mega X to build up phylogenetic trees. In order to eliminate the influences of different algorithms of constructing trees, we apply the Unweighted Pair Group Method with Arithmetic Mean (UPGMA) algorithm (Sneath and Sokal, 1973) for analysis on the four datasets.

For comparison with other common alignment or alignment-free method, we also perform *k-mer* and MSA (ClustalW or MUSCLE) on the same dataset. The Feature frequency profile (FFP) (Woo et al., 2005), which is based on *k-mer* frequency, calculates the frequency of each *k-mer* in the sequence and turns a DNA sequence into a vector in a 4^*k*^-dimensional space. The Euclidean distance between two *k-mer* vectors can also be computed by formula (17). We apply MSA method, ClustalW on several datasets as well, with the default parameters in Mega X. ClustalW is much slower than another MSA algorithm, MUSCLE, while ClustalW can give a better result. MUSCLE is applied on the fourth dataset of 351 viruses and after we get the alignment result of the viruses, distance matrix is calculated using Hamming distance, to find the nearest neighbor of each virus. Hamming distance between two strings of equal length is the number of positions at which the corresponding symbols are different. It measures the minimum number of substitutions required to change one string into the other or the minimum number of errors that could have transformed one string into the other. Since alignment approaches are to arrange the sequences to identify regions of similarity between the sequences, the alignment would provide the performance of each sequence on a fixed number of positions. Therefore, the Hamming distance can be calculated by simply counting the number of pairwise differences in character states.

In the simulated dataset, we use the pairwise alignment distance by the “seqpdist” function inside MATLAB Bioinformatics toolbox, which uses the Jukes-Cantor algorithms as the correct tree, since the sequences are simulated according to a base sequence. Then the distance matrices are compared using Robinson-Foulds distances, which can measure the congruence to the reference topology.

## Results

We apply the Accumulated Natural Vector method on five datasets, and compare the results with common methods, such as MSA, *k-mer* (FFP) and the traditional Natural Vector method. From comparison, the results of Accumulated Natural Vector are more accurate and the calculation cost is very small compared to others. A dataset of 351 viruses has also been tested, and laptop cannot bear such a heavy burden of calculation of aligning them but alignment-free can still be done in a reasonable time. We also use a server to align segments of 351 sequences, to compare the results to ANV and other methods. ANV also gives the best performance on this dataset. Besides, we simulate another dataset of 20 sequences from a randomly generated sequence with length of 1,000bp, and test the phylogenetic trees from this and other methods.

We have chosen those datasets of different sizes (number of sequences, and lengths of sequences), which to test if ANV can be suitable in all cases. Most datasets have been analyzed by previous researches, therefore we can compare our results to others to evaluate the performances. Four datasets consist of viruses that are closely related to human health, and the mammal's dataset and simulated dataset show that this method can perform on other types of sequences as well.

### Coronaviruses Dataset

**Figure 1:**
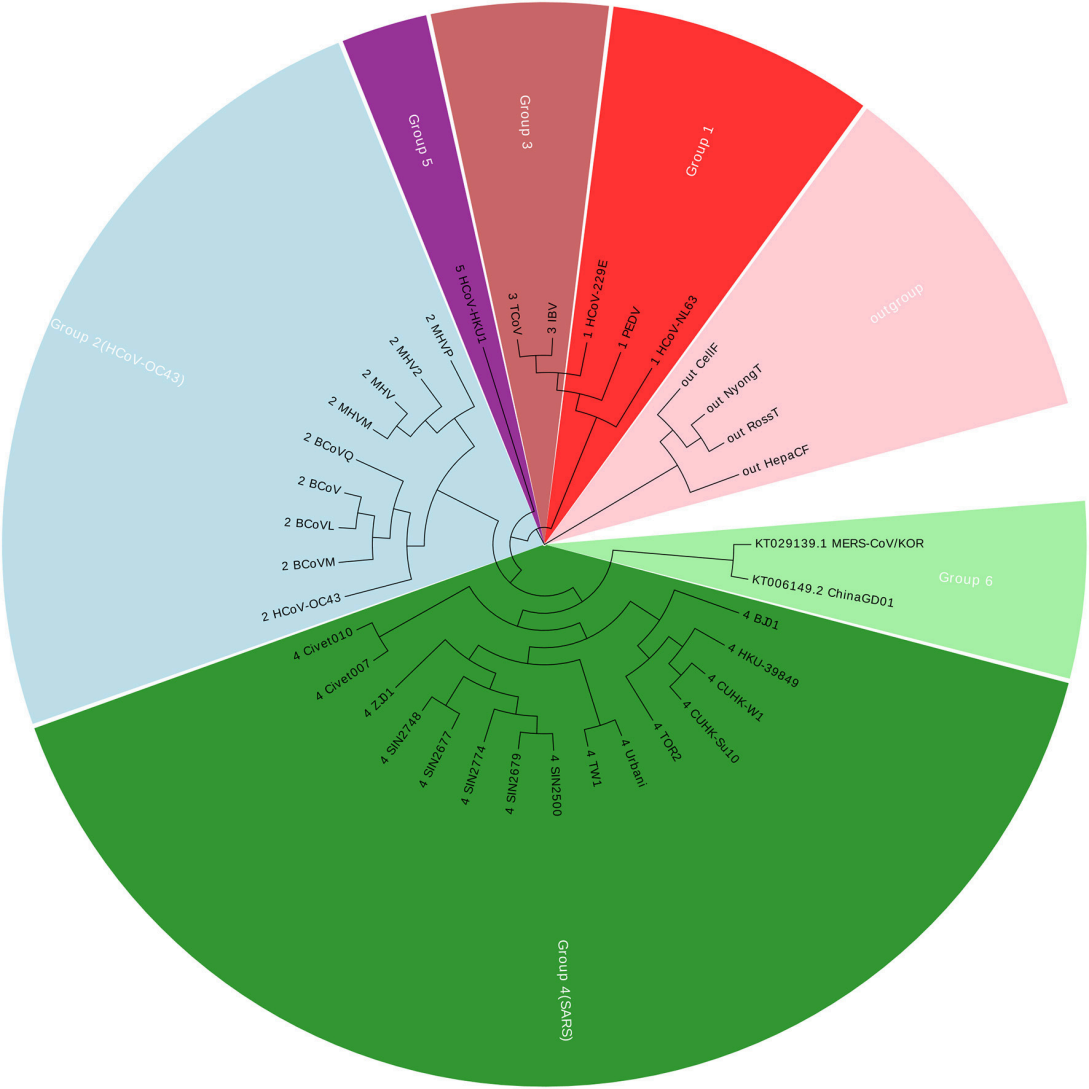
The phylogenetic UPGMA tree using ANV method on Coronaviruses dataset.

Coronavirus belongs to the subfamily Coronavirinae in the family Coronaviridae, in the order Nidovirales. In this paper, we construct a dataset with 36 Coronaviruses, in which 34 viruses are from the exact same dataset with (Woo et al., 2005; Yu et al., 2010; Hoang et al., 2015). The other two viruses are two new members in Coronavirus. Details of the Coronaviruses can be found in Table S1. The new ChinaGD01 (Lu et al., 2015) was identified in Guangdong Province (China) in 2015 and is an imported Middle East respiratory syndrome Coronavirus. The other one MERS-CoV/KOR is from South Korea (Kim et al., 2015). As of 15 June 2015, the MERS-COV was spreading in South Korea, and the ChinaGD01 case was a South Korean national who traveled to Guangdong in May 2015. Therefore, those two members were considered highly correlated with each other. The genomic size of Coronaviruses ranges from about 9 to 31 kbp, with the average of 27,567 nucleotides. Using our Accumulated Natural Vector and UPGMA method (Sneath and Sokal, 1973), we can build up a phylogenetic tree as shown in Figure 1.

Figure 1 shows that the two new members are clustered together with Group 4, which is also well-known as SARS (Severe Acute Respiratory Syndrome). Between November 2002 and July 2003, an outbreak of SARS in southern China caused an eventual 8,098 cases, resulting in 774 deaths reported in 37 countries. Both MERS-CoV and SARS viruses are beta-Coronaviruses, however, they belong to different lineages, for more details please see (Drexler et al., 2013; Hilgenfeld and Peiris, 2013). The phylogenetic tree indicates that the ChinaGD01 and MERS-CoV/KOR forms a monophyletic clade, sister to the SARS clade, which may possibly be a variant from some SARS viruses.

We also performed the same procedure with *k-mer* method on the Coronaviruses dataset. However, how to choose an optimal *k*-value is an interesting topic that requires manual intervention. Sims et al. showed in Woo et al. (2005) that the location of the peak in the distribution of k-mers, i.e., the k with the largest vocabulary, is related to the sequence length N. The k with maximum information is empirically determined but may be closely approximated by

(18) $$\begin{array}{l} k_{Hmax}=\log_{4}N \end{array}$$

where 4 is the alphabet size. They have shown in Sims et al. (2009) that reliable tree topologies are typically obtained with *k-mer* resolutions where k > *k*_*Hmax*_ whereas lengths below *k*_*H*max_ yield unreliable trees. The upper limit of resolution can be empirically determined by a criterion that the tree topology for feature length k is equal to that of k+1, i.e., tree topologies converge.

**Figure 2:**
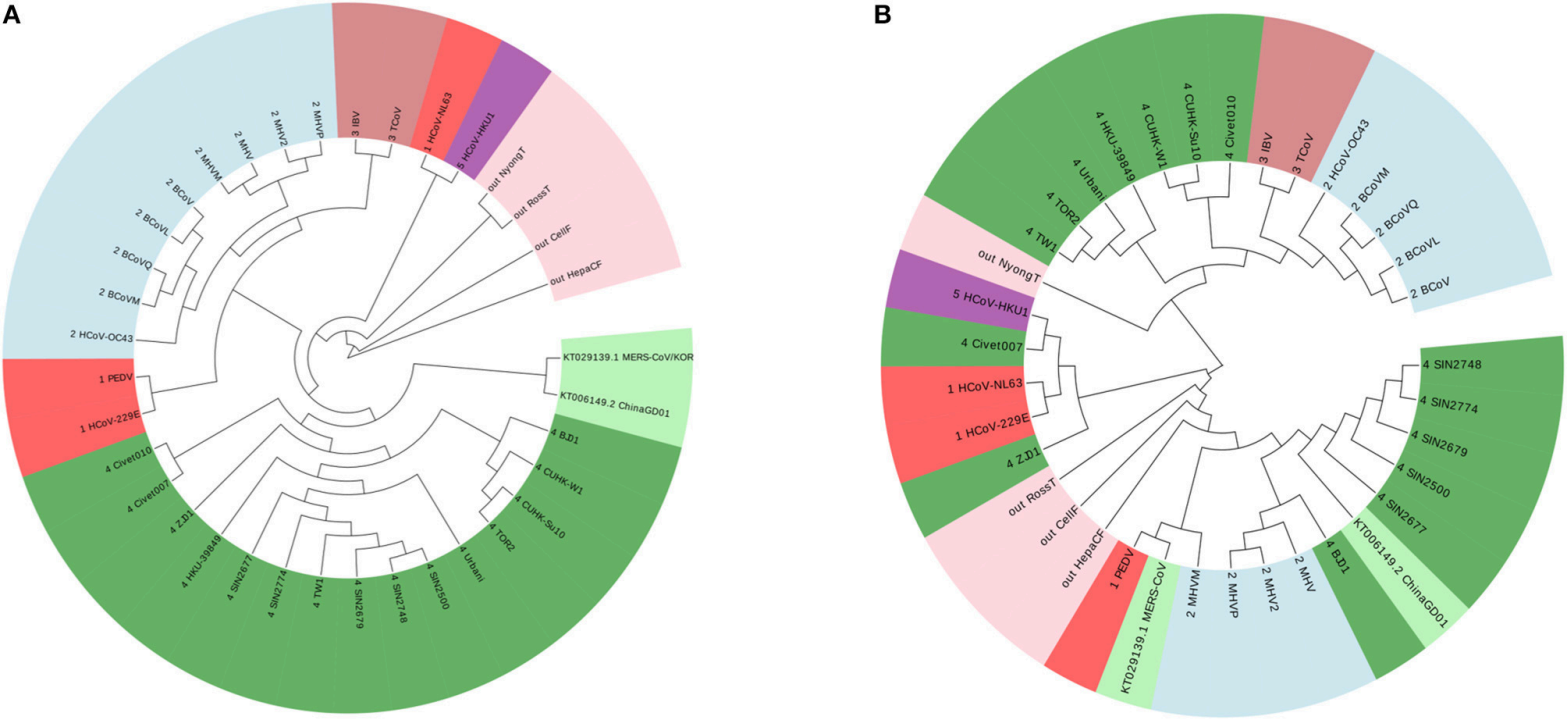
**(A)** The phylogenetic UPGMA tree using FFP (*k-mer*) method when on Coronavirus dataset. **(B)** The phylogenetic UPGMA tree using MSA (ClustalW) method on Coronaviruses dataset.

According to this principle, we have 7 ≤ k ≤ 9. We show the result of *k* = 7 in Figure 2A, and the results of *k* = 8 and *k* = 9 are in the Figures S1, S2. The four outgroup viruses cannot be clustered together as another branch from the tree of Coronaviruses, meanwhile the Group 1 was divided into smaller groups. The traditional ClustalW algorithm of Multiple Sequence Alignment (MSA) is also applied on the same dataset, and the result is shown in Figure 2B. MSA cannot cluster viruses from same groups together either. From this example, we can see that our ANV method is better than the *k-mer* and MSA method.

### Influenza A viruses

**Figure 3:**
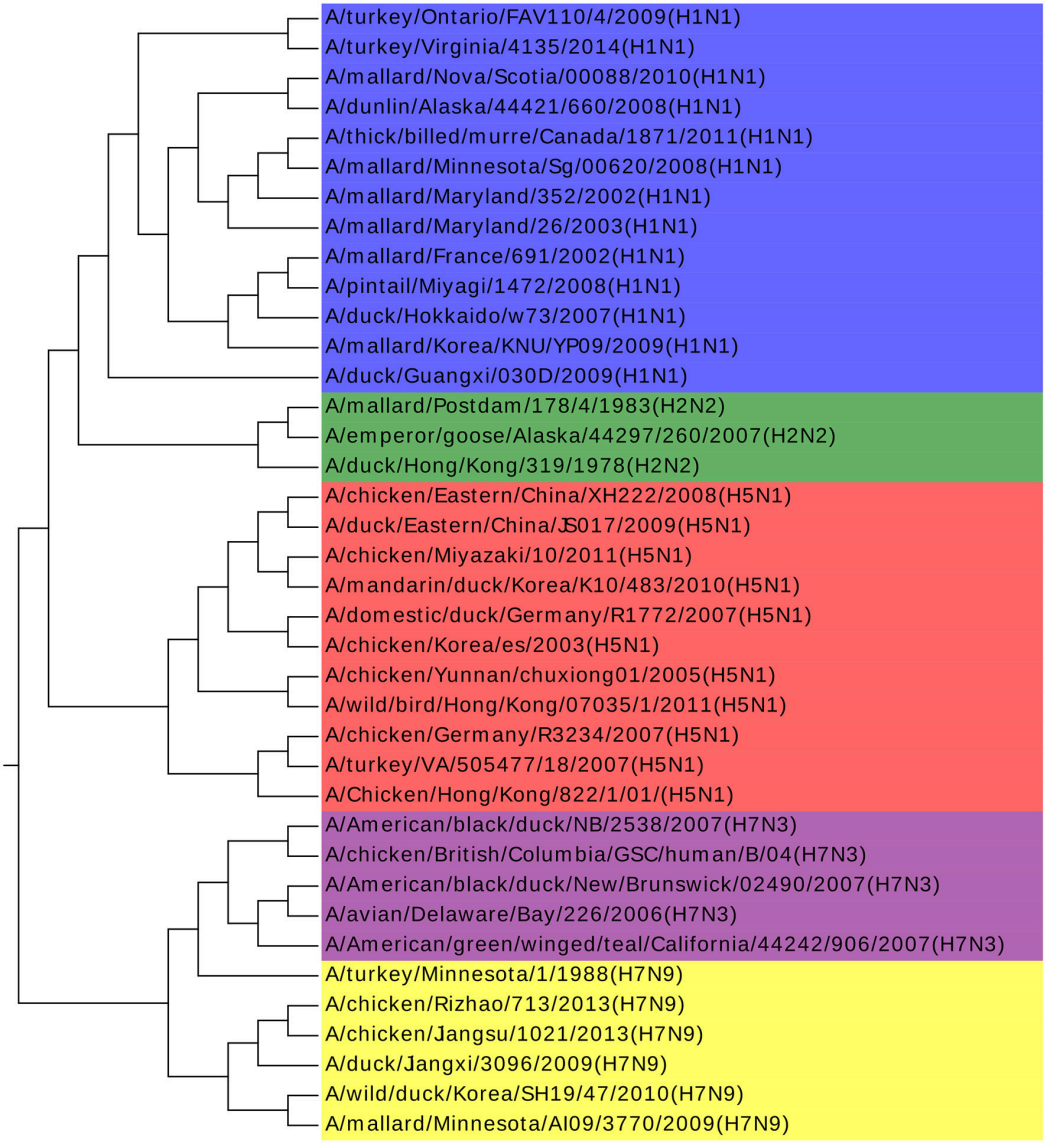
The phylogenetic UPGMA tree using ANV method on Influenza A viruses dataset.

Influenza A viruses are single-stranded RNA viruses, which have been a major health threat to both human society and animals (Hoang et al., 2015). Influenza A viruses' nomenclature is based on the surface glycoproteins: hemagglutinin (HA) and neuraminidase (NA) (Obenauer et al., 2006). HA has 15 subtypes and NA has 9 subtypes, which forms 135 different combinations. The NCBI number of the analyzed 38 Influenza A viruses can be found in Table S2. Our result agrees with previous work by Hoang et al. (2015). Furthermore, we find that all the Influenza A viruses are clustered with the same H and N type in Figure 3, with only one exception of A/turkey/Minnesota/1/1988(H7N9). There is no specific research about this virus and we infer that it may be the intermediate from H7N3 to H7N9. H7N3 had an outbreak in July 2012, causing millions of poultry's infection, but there is no report of infection from human to human yet. However, H7N9 was identified in Shanghai, China at the end of March 2013. Considering that the HA glycoprotein of those two subtypes are the same and the close outbreak date, we indicate that the H7N9 on March 2013 might be a variant from H7N3, and A/turkey/Minnesota/1/1988 (H7N9) plays a key role in this variation. We get the same conclusion in another work as well (Dong et al., 2018). More biological research on this virus should be done to deepen our understanding of Influenza A viruses to accelerate the invention of an effective vaccine and to prevent more dangerous variants.

**Figure 4:**
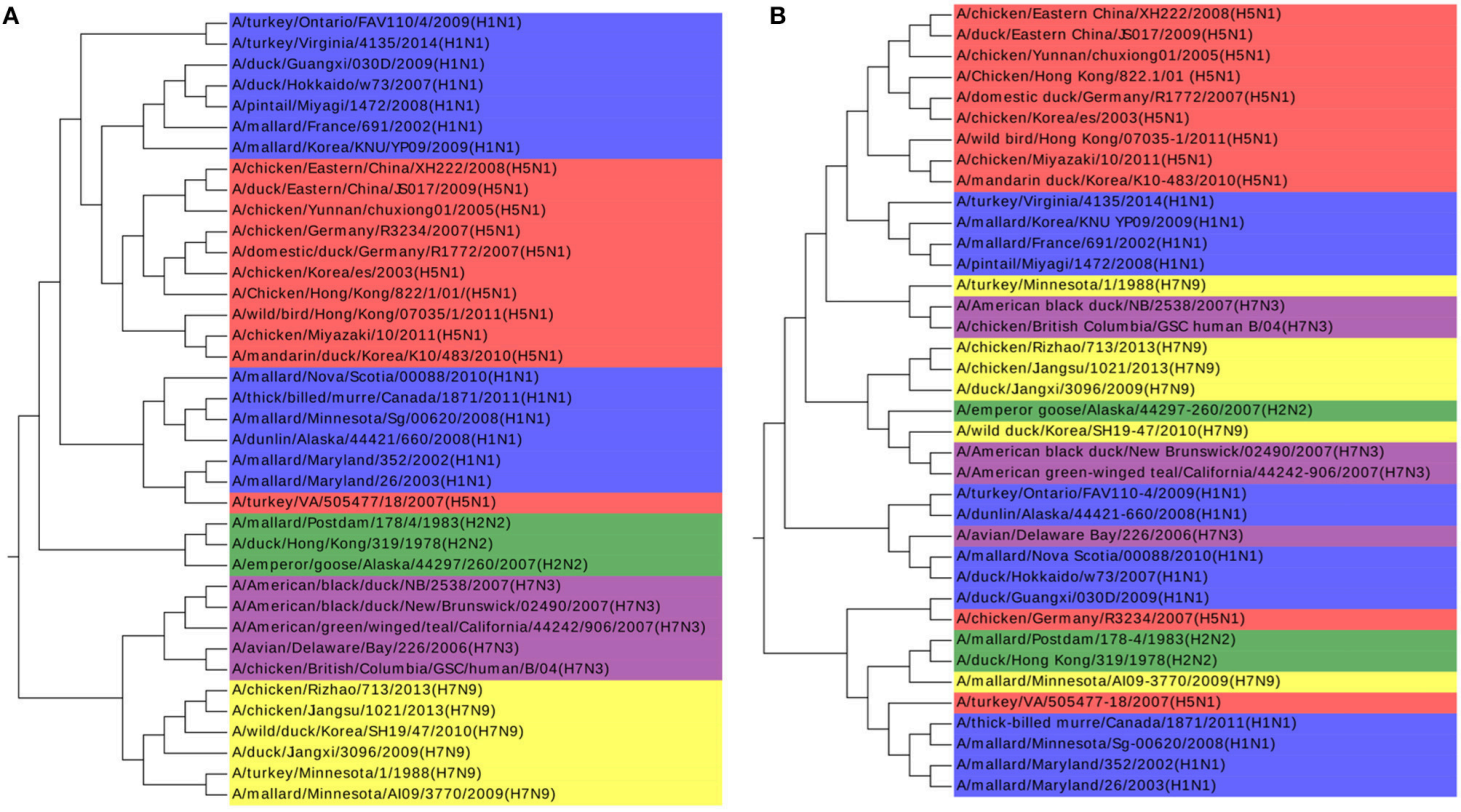
**(A)** The phylogenetic UPGMA tree using the *k-mer* (*k* = 5) method on Influenza A viruses dataset. **(B)** The phylogenetic UPGMA tree using MSA (ClustalW) method on Influenza A viruses dataset.

The *k-mer* method and MSA are also performed on this dataset as shown in Figures 4A,B. The *k*-value is determined in the same procedure as in the Coronaviruses dataset as 5. In Figure 4A, the viruses from H1N1 and H5N1 are mixed up together with each other, while MSA has a worse result in Figure 4B. The results also indicate that *k-mer* and MSA cannot reveal the real relationships among the viruses.

**Figure 5:**
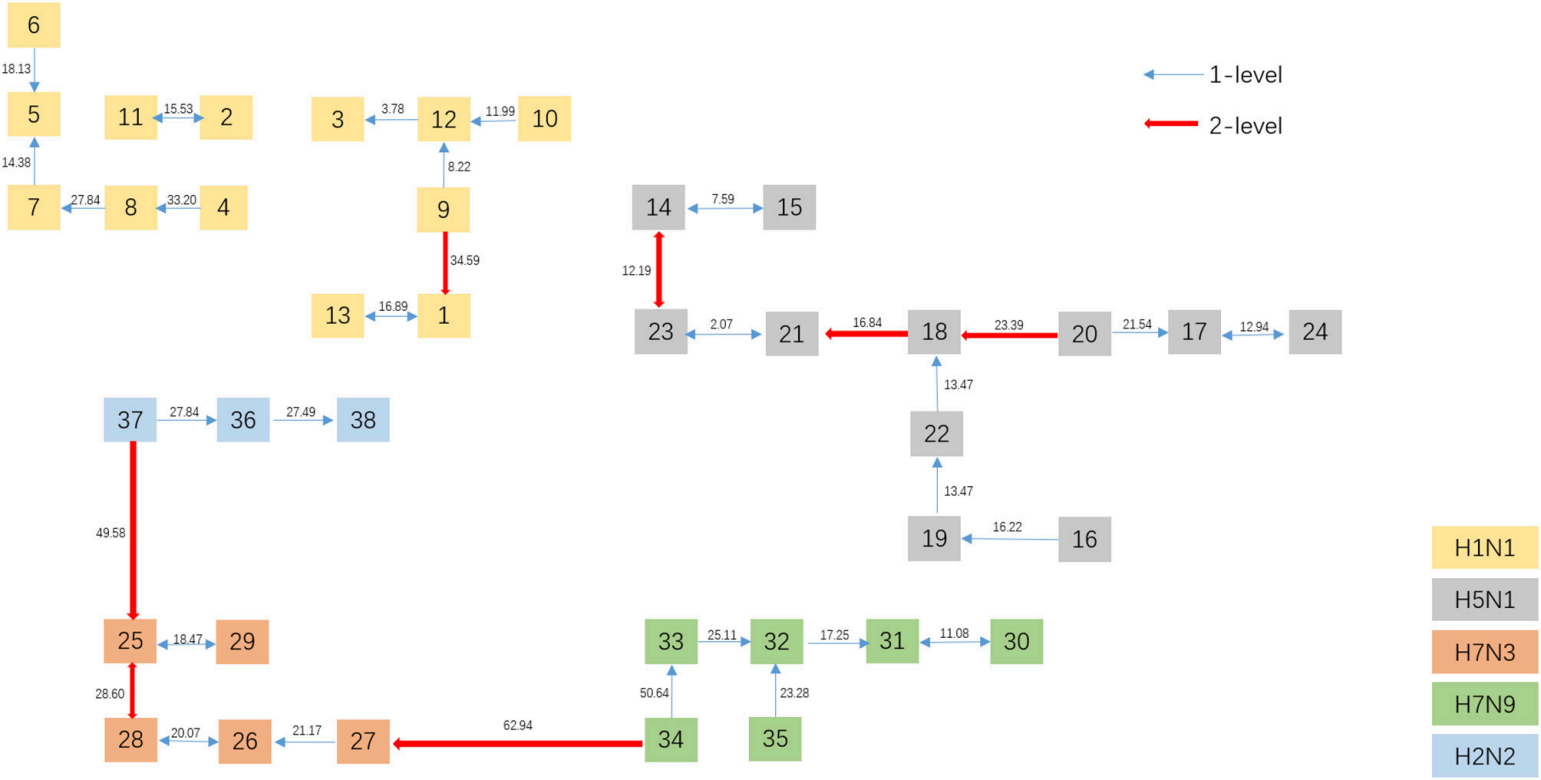
The Natural Graph using ANV method on Influenza A virus dataset.

To get a direct image of the relationships between Influenza A viruses, we draw the Natural Graph of them. Natural Graph was first introduced by Zheng et al. (2015). In Figure 5, the blue lines represent the 1-level connected components and the red ones 2-level. Classes are marked in different colors and it is obvious that after the construction of two levels, the Influenza A viruses with the same H and N are clustered together, including the A/turkey/Minnesota/1/1988(H7N9) which is Number 34 in Figure 3. H7N9 and H7N3 are clustered together in Level 2, indicating that they have a closer relationship, which accords with our previous conjecture.

### 72 Ebolaviruses Dataset

**Figure 6:**
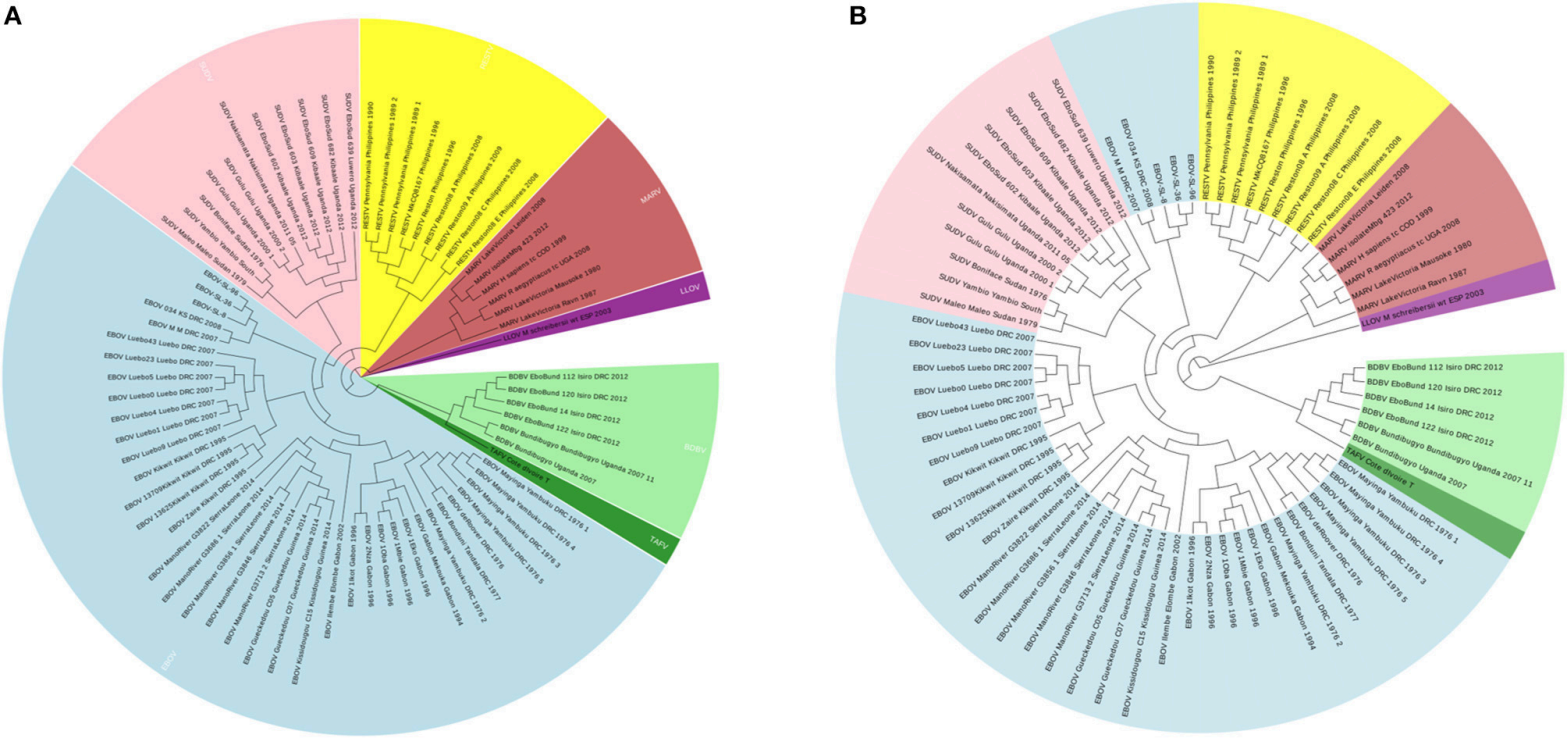
**(A)** The phylogenetic UPGMA tree using ANV method on Ebolaviruses dataset. **(B)** The phylogenetic UPGMA tree using the traditional Natural Vector (NV) method on Ebolaviruses dataset.

To illustrate that the new proposed ANV method is an important improvement of the traditional Natural Vector method, a 72 Ebolaviruses dataset is tested, which is a subset of the 163 viruses used in Zheng et al. (2015). It consists of 38 Ebola virus (EBOV), 11 Sudan virus (SUDV), 9 Reston virus (RESTV), 1 Taï Forest virus (TAFV), 6 Bundibugyo virus (BDBV), 6 Marburg virus (MARV) and 1 Lloviuvirus (LLOV). Details of this dataset are shown in Table S3. In Figure 6A, the phylogenetic tree shows that from the novel Accumulated Natural Vector method classifies all viruses into the right groups, however, in Figure 6B, the traditional Natural Vector method divides EBOV class into two clusters and SUDV is misclassified with some EBOV virus. This is an indication that including covariance between nucleotides helps improve the accuracy of classification. Hence this is an important improvement to the traditional Natural Vector and other alignment-free methods.

### 351 Viruses Dataset

We also test a large dataset of 351 viruses in Li et al. (2016), and the details of this dataset can be found in Table S4. The average length of them is 11,952 nucleotides and it makes alignment methods on a laptop impossible. Only server or cloud computing can finish such a task. Here we use 1-Nearest Neighbor (1-NN) method (Li et al., 2016) to see the accuracy of the prediction. This evaluation is inspired by the high rate of missing labels in many databases of viruses. For example, if a virus with missing family label has been added to the database, and it should share the same family label with the virus (stored in the database already) that is closest to it, then we can predict the missing family label according to the information of its nearest neighbor. Therefore, for a dataset with no missing labels, we can count how many viruses share the same label with its neighbor. “Nearest neighbor” of a specific virus can be defined as the virus that has the smallest Euclidean distance in the dataset to it for the alignment-free methods. For alignment results, we use the Hamming distance to measure the distance between two sequences. If the virus shares its distance with its neighbor, we consider it as a “correct” one, since even if its label is missing we can still predict it from its nearest neighbor. The accuracy can be computed by dividing the number of correct ones by the number of all viruses, in this case, by 351. We compare the result of ANV to the *k-mer* method since they are all alignment-free methods, and the results are shown in Table 3. The optimal choice of k is made by the same procedure in the other datasets. From Table 3, it is evident that ANV has much higher accuracy than the *k-mer* method, meanwhile using much less time. Thus, we have proved that ANV can apply to practical use with high time-efficiency and high-accuracy. For the alignment in this part, we tried to align all the sequences with full length on our server, but it fails to give a reliable result. Therefore, we extract 3,000 bp from the beginning and align 351 pieces of segments all with length of 3,000 bp. The results are shown in Table 3 as well and the accuracy is still not as good as what ANV gives.

**Table 3:** Comparison of ANV and *k-mer* methods on 351 viruses dataset.

**METHOD**	**ANV**	**6-MER**	**7-MER**	**8-MER**	**MSA (MUSCLE)**
Family	94.87%	71.23%	25.36%	16.24%	72.08%
Genus	83.19%	65.24%	21.65%	12.25%	65.53%
Computing Time (seconds)	2466.73	4179.24	8636.13	24011.70	Unable to compute on laptop

### Mammals

Our Accumulated Natural Vector performs well not only on virus datasets, but also on other common species. We extract 31 mammalian mitochondrial genomes with the average length of 16,696 nucleotides, and the NCBI numbers of them can be found in Table S5. The genomes are from seven known clusters: Primates, Carnivora, Cetartiodactyla, Perissodactyla, Eulipotyphla, Lagomorpha, and Rodentia.

**Figure 7:**
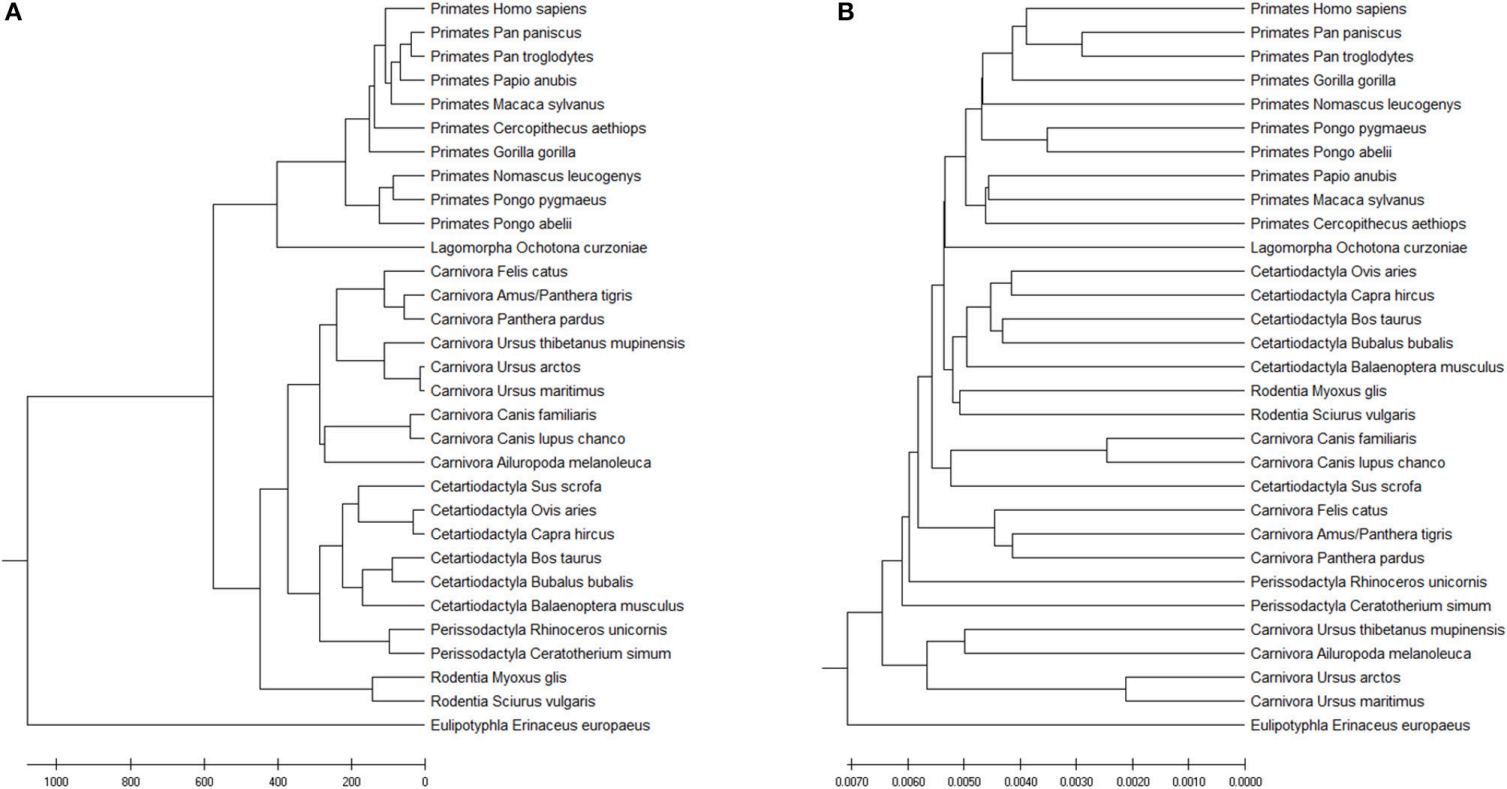
**(A)** The phylogenetic UPGMA tree using ANV method on mammals mtDNA dataset. **(B)** The phylogenetic UPGMA tree using FFP (*k-mer*) method when K = 8 on mammals mtDNA dataset.

The Accumulated Natural Vector method can still distinguish the differences among the seven clusters, as shown in Figure 7A. FFP (*k-mer*) method has also been tested as well (the optimal *k*-value for this dataset is 8), as shown in Figure 7B. Since the species that includes in different paper are not all the same, it is hard to compare the whole topology of phylogenetic trees, however, our work still only has a small difference from the previous work in Murphy et al. (2001) and Tarver et al. (2016). The difference can be attributed to that mitochondrial genomes in mammals may not always reflect the organismal evolutionary history (Morgan et al., 2014), however, it still keeps more information than *k-mer* does in Figure 7B, since the distance within each group is smaller than the distances among groups, we can still distinguish clusters based on current dataset. In Ladoukakis and Zouros (2017), point out that most of the information researchers gained about the tree of life through the use of mtDNA remains valid, while we should pay more attention to its role in the function of the organism and its value as a tool in the study of major evolutionary novelties in the history of life. Therefore, the result implies that our ANV method can capture the key information hidden inside the DNA sequences and gives us a reliable topology among mammals.

### Simulated Dataset

**Figure 8:**
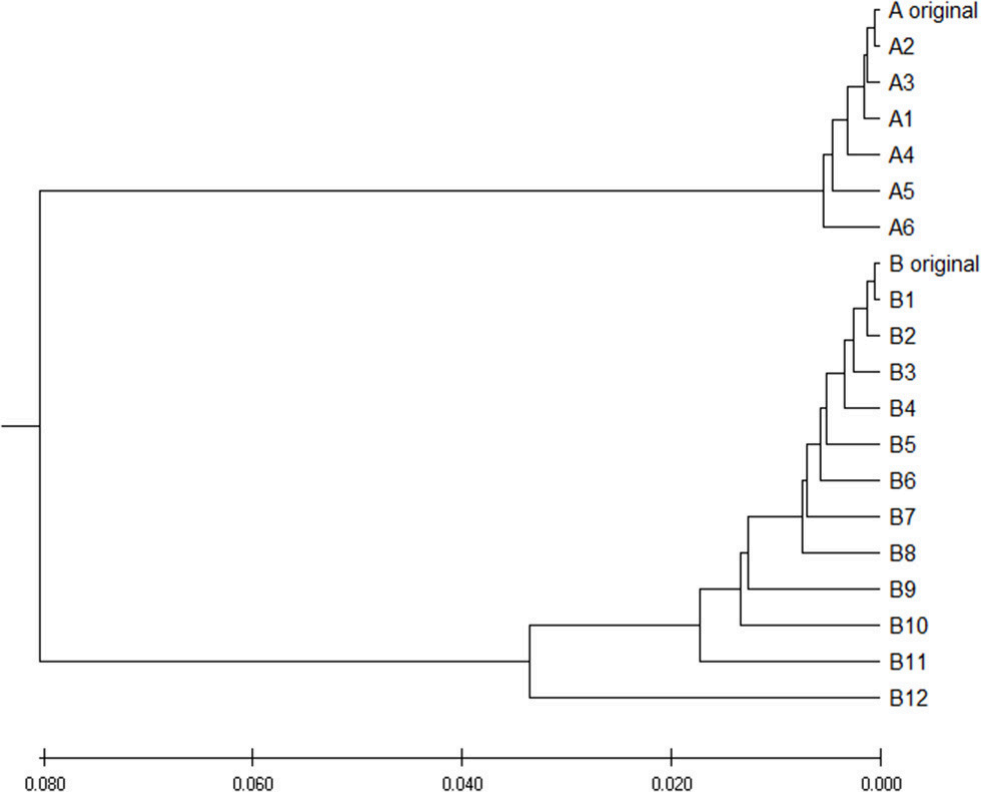
The phylogenetic UPGMA tree using Jukes-Cantor pairwise alignment method on simulated dataset.

**Figure 9:**
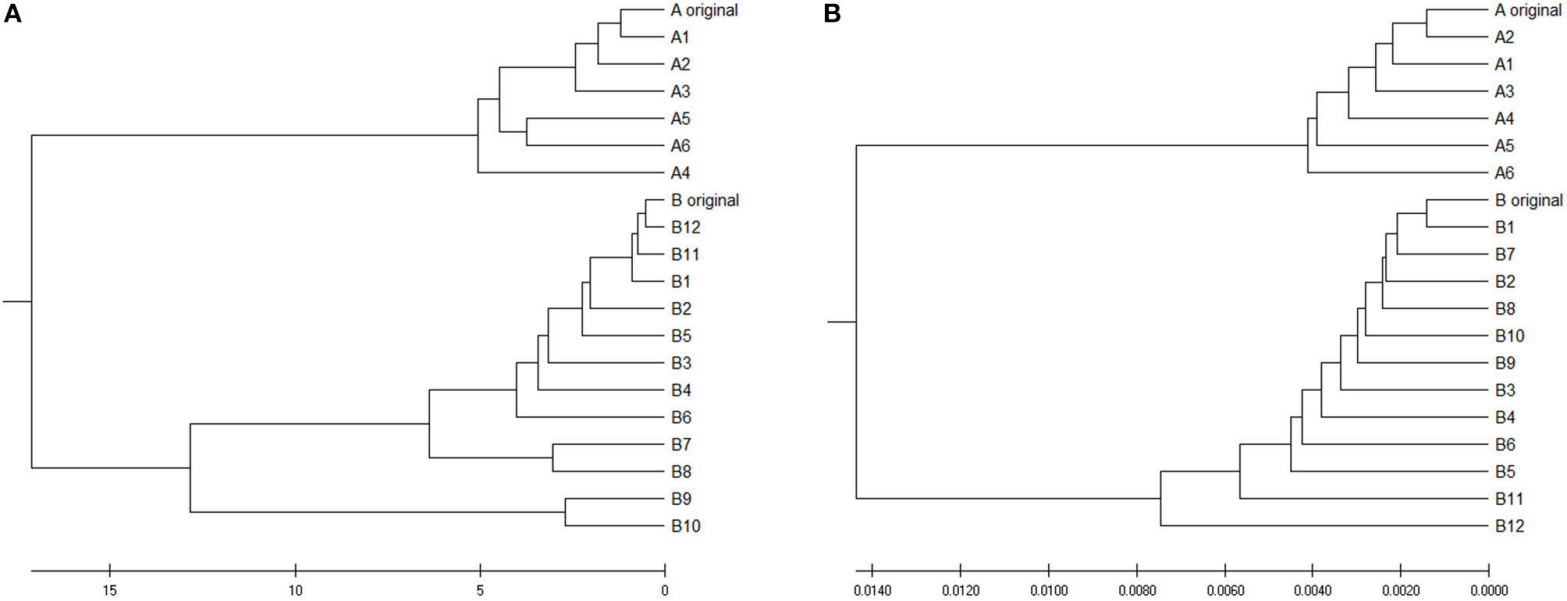
**(A)** The phylogenetic UPGMA tree using ANV method on simulated dataset **(B)** The phylogenetic UPGMA tree using FFP (*k-mer*) method when K = 4 on simulated dataset.

To verify is the similarity distance by our method can be used for clustering DNA sequences effectively, we also generated different mutations in DNA sequences and constructed phylogenetic trees by various methods. We simulated a sequence of length 1,000 bp as a base sequence, and generated two new sequences named “A_original” and “B_original” using point mutations. Both A and B have 100 nucleotides different from the original sequence. We then similarly evolved A and B into different mutants by four different mutations (substitutions, deletion, insertion, and transposition) as did in Yin et al. (2014). Table 4 is the detailed description on the simulated DNA sequences with different mutations. Since the sequences are mutated slightly based on an exon sequence, we take the aligned result as the “correct” relationships among the sequences, and the alignment is done by the “seqpdist” function in MATLAB Bioinformatics toolbox. This function uses the classical Jukes-Cantor algorithm and we calculate the pairwise alignment distance. For comparison, we use the ANV method, FFP method (we test *k* = 4,5,6 in this case, since the lengths of sequences are about 1,000 bp). The UPGMA trees of alignment, ANV and FFP (k = 4) methods are shown in Figures 8, 9A,B separately. Among these trees, it is not very obvious which one is more similar to the alignment results, therefore we calculate the Robinson-Foulds distances between the distance matrix and the “correct” matrix and the results are shown in Table 5. Here we apply the program named “Robinson-Foulds” (Robinson and Foulds, 1981) when calculating Table 5. The simulated dataset is in Table S6. Actually, the differences among trees mainly lie in the branch of sequences generated from B, and ANV gives a more similar result, since the order is slightly disorganized by B5 and the transpositional sequences, while in Figure 9B, the whole branch of B is different from the alignment result.

**Table 4:** Description of DNA sequence mutation in simulated tests.

**Sequence Name**	**Description**
A_original	200 point mutations from the randomly generated sequence with length 1,000 bp
A1	2 random nucleotide substitutions in A
A2	2 random nucleotide substitutions in A
A3	5 random nucleotide substitutions in A
A4	5 random nucleotide substitutions in A
A5	10 random nucleotide substitutions in A
A6	10 random nucleotide substitutions in A
B_original	200 point mutations from the randomly generated sequence with length 1,000 bp (different from A_original)
B1	2 random nucleotide substitutions in B_original
B2	2 random nucleotide substitutions in B_original
B3	5 random nucleotide substitutions in B_original
B4	5 random nucleotide substitutions in B_original
B5	10 random nucleotide substitutions in B_original
B6	10 random nucleotide substitutions in B_original
B7	10 bp Deletion from positions 51:60 in B_original
B8	10 bp Deletion from positions 601:610 in B_original
B9	20 bp Insertion at position 51 in B_original
B10	20 bp Insertion at position 601 in B_original
B11	50 bp Transposition from position 1 to 50 in B_original
B12	100 bp Transposition from position 601 to 700 in B_original

**Table 5:** Robinson-Foulds distances between trees by alignment-free methods and the reliable alignment tree.

**Method**	**Alignment**	**ANV**	**4-mer**	**5-mer**	**6-mer**
distance	0	23	27	29	29

## Discussion

In this paper, we propose a novel vector named Accumulated Natural Vector to analyze sequences, genomes and their phylogenetic relationships. Results from our analysis largely agree with the earlier studies, which indicates that our approach can detect the similarity and difference among sequences. Therefore, constructing phylogenetic trees only by sequence data could be done accurately in a very reasonable time, without using large computing platforms or conducting biological experiments of high cost. Our method can be applied in a global comparison of all genomes and provide a new powerful tool by including the correlations of nucleotides. We are working on extending the ANV method to protein sequences, nevertheless, for a protein sequence, it would produce an 1,830-dim vector for each sequence. The calculation cost for this is too large under the current technology. The covariance for three amino acids at a time may be more reasonable, since three consequent nucleotides can also become a codon in expression region of a sequence.

## Author Contributions

SS-TY and RH conceived the idea of covariance. RD implemented the idea and wrote the first draft of the manuscript. LH discussed and revised the first draft. RD, LH, RH, and SS-TY all contributed to the writing of the manuscript and agreed with the manuscript results and conclusions. They jointly developed the structure and arguments for the paper, made critical revisions and approved final version, and reviewed and approved the final manuscript.

### Conflict of Interest Statement

The authors declare that the research was conducted in the absence of any commercial or financial relationships that could be construed as a potential conflict of interest.
