# Supplementary material for: A Novel Approach to Clustering Genome Sequences Using Inter-nucleotide Covariance
Source: Front Genet. 2019 Apr 9;10:234. doi: 10.3389/fgene.2019.00234 (PMC6465635; doi:10.3389/fgene.2019.00234)
Supplement: Supplementary file 1 [file Data_Sheet_1.docx]

Supplementary Material

A Novel Approach to Clustering Genome Sequences

Using Inter-nucleotide Covariance

Rui Dong^1^, Lily He^1^, Rong Lucy He^2^, Stephen S.-T. Yau^1^

*** Correspondence:** Prof. Stephen S.-T. Yau: yau@uic.edu

# Supplementary Data

**Table S1. Information from GenBank of the 36 *Coronaviruses***

| Accession  Number | Abbreviation | Length  (nt) | Description/Classification |
| --- | --- | --- | --- |
| AF304460 | HCoV-229E | 27317 | Human coronavirus 229E, complete genome/Group 1 |
| AY391777 | HCoV-OC43 | 30738 | Human coronavirus OC43, complete genome/Group 2 |
| AF353511 | PEDV | 28033 | Porcine epidemic diarrhea virus strain, complete genome/Group 1 |
| U00735 | BCoVM | 31032 | Bovine coronavirus strain Mebus, complete genome/Group 2 |
| AF391542 | BCoVL | 31028 | Bovine coronavirus isolate BCoV-LUN, complete genome/Group 2 |
| AF220295 | BCoVQ | 31100 | Bovine coronavirus strain Quebec, complete genome/Group 2 |
| NC_003045 | BCoV | 31028 | Bovine coronavirus, complete genome/Group 2 |
| AF208067 | MHVM | 31233 | Murine hepatitis virus strain ML-10, complete genome/Group 2 |
| AF201929 | MHV2 | 31276 | Murine hepatitis virus strain 2, complete genome/Group 2 |
| AF208066 | MHVP | 31112 | Murine hepatitis virus strain Penn 97-1, complete genome/Group 2 |
| NC_001846 | MHV | 31357 | Murine hepatitis virus, complete genome/Group 2 |
| NC_001451 | IBV | 27608 | Avian infectious bronchitis virus, complete genome/Group 3 |
| EU095850 | TCoV | 27657 | Turkey coronavirus isolate MG10, complete genome/Group 3 |
| AY278488 | BJ01 | 29725 | SARS coronavirus BJ01, complete genome/Group 4 |
| AY278741 | Urbani | 29727 | SARS coronavirus Urbani, complete genome/Group 4 |
| AY278491 | HKU-39849 | 29742 | SARS coronavirus HKU-39849, complete genome/Group 4 |
| AY278554 | CUHK-W1 | 29736 | SARS coronavirus CUHK-W1, complete genome/Group 4 |
| AY282752 | CUHK-Su10 | 29736 | SARS coronavirus CUHK-Su10, complete genome/Group 4 |
| AY283794 | SIN2500 | 29711 | SARS coronavirus isolate SIN2500, complete genome/Group 4 |
| AY283795 | SIN2677 | 29705 | SARS coronavirus isolate SIN2677, complete genome/Group 4 |
| AY283796 | SIN2679 | 29711 | SARS coronavirus isolate SIN2679, complete genome/Group 4 |
| AY283797 | SIN2748 | 29706 | SARS coronavirus isolate SIN2748, complete genome/Group 4 |
| AY283798 | SIN2774 | 29711 | SARS coronavirus isolate SIN2774, complete genome/Group 4 |
| AY291451 | TW1 | 29729 | SARS coronavirus TW1, complete genome/Group 4 |
| NC_004718 | TOR2 | 29751 | SARS coronavirus TOR2, complete genome/Group 4 |
| AY297028 | ZJ01 | 29715 | SARS coronavirus ZJ01, complete genome/Group 4 |
| AY572034 | Civet007 | 29540 | SARS coronavirus civet007, complete genome/Group 4 |
| AY572035 | Civet010 | 29518 | SARS coronavirus civet010, complete genome/Group 4 |
| NC_005831 | HCoV-NL63 | 27553 | Human coronavirus NL63, complete genome/Group 1 |
| NC_006577 | HCoV-HKU1 | 29926 | Human coronavirus HKU1, complete genome/Group 5 |
| NC_001564 | CellF | 10695 | Cell fusing agent virus, complete genome/Flaviviridae (outgroup) |
| NC_004102 | HepaCF | 9646 | Hepatitis C virus, complete genome/Flaviviridae (outgroup) |
| NC_001512 | NyongT | 11835 | O’nyong-nyong virus, complete genome/Togaviridae (outgroup) |
| NC_001544 | RossT | 11657 | Ross River virus, complete genome/Togaviridae (outgroup) |
| KT029139 | MERS-CoV/KOR | 30108 | Middle East respiratory syndrome coronavirus isolate |
| KT006149 | ChinaGD01 | 30114 | Middle East respiratory syndrome coronavirus strain ChinaGD01 |

**Table S2. Information from GenBank of the 38 Influenza A viruses**

| Accession  Number | Length  (nt) | Description |
| --- | --- | --- |
| HM370969 | 1419 | A/turkey/Ontario/FAV110-4/2009(H1N1) |
| CY138562 | 1422 | A/mallard/Nova Scotia/00088/2010(H1N1) |
| CY149630 | 1433 | A/thick-billed murre/Canada/1871/2011(H1N1) |
| KC608160 | 1398 | A/duck/Guangxi/030D/2009(H1N1) |
| AM157358 | 1413 | A/mallard/France/691/2002(H1N1) |
| AB470663 | 1422 | A/duck/Hokkaido/w73/2007(H1N1) |
| AB546159 | 1410 | A/pintail/Miyagi/1472/2008(H1N1) |
| HQ897966 | 1410 | A/mallard/Korea/KNU YP09/2009(H1N1) |
| EU026046 | 1433 | A/mallard/Maryland/352/2002(H1N1) |
| FJ357114 | 1433 | A/mallard/Maryland/26/2003(H1N1) |
| GQ411894 | 1413 | A/dunlin/Alaska/44421-660/2008(H1N1) |
| CY140047 | 1433 | A/mallard/Minnesota/Sg-00620/2008(H1N1) |
| KM244078 | 1410 | A/turkey/Virginia/4135/2014(H1N1) |
| HQ185381 | 1350 | A/chicken/Eastern China/XH222/2008(H5N1) |
| HQ185383 | 1350 | A/duck/Eastern China/JS017/2009(H5N1) |
| EU635875 | 1350 | A/chicken/Yunnan/chuxiong01/2005(H5N1) |
| FM177121 | 1370 | A/chicken/Germany/R3234/2007(H5N1) |
| AM914017 | 1350 | A/domestic duck/Germany/R1772/2007(H5N1) |
| KF572435 | 1350 | A/wild bird/Hong Kong/07035-1/2011(H5N1) |
| AF509102 | 1366 | A/Chicken/Hong Kong/822.1/01 (H5N1) |
| AB684161 | 1350 | A/chicken/Miyazaki/10/2011(H5N1) |
| EF541464 | 1350 | A/chicken/Korea/es/2003(H5N1) |
| JF699677 | 1350 | A/mandarin duck/Korea/K10-483/2010(H5N1) |
| GU186511 | 1370 | A/turkey/VA/505477-18/2007(H5N1) |
| EU500854 | 1453 | A/American black duck/NB/2538/2007(H7N3) |
| CY129336 | 1428 | A/American black duck/New Brunswick/02490/2007(H7N3) |
| CY076231 | 1420 | A/American green-winged teal/California/44242-906/2007(H7N3) |
| CY039321 | 1434 | A/avian/Delaware Bay/226/2006(H7N3) |
| AY646080 | 1453 | A/chicken/British Columbia/GSC_human_B/04(H7N3) |
| KF259734 | 1398 | A/chicken/Rizhao/713/2013(H7N9) |
| KF938945 | 1404 | A/chicken/Jiangsu/1021/2013(H7N9) |
| KF259688 | 1413 | A/duck/Jiangxi/3096/2009(H7N9) |
| KC609801 | 1426 | A/wild duck/Korea/SH19-47/2010(H7N9) |
| CY014788 | 1460 | A/turkey/Minnesota/1/1988(H7N9) |
| CY186004 | 1422 | A/mallard/Minnesota/AI09-3770/2009(H7N9) |
| DQ017487 | 1467 | A/mallard/Postdam/178-4/1983(H2N2) |
| [CY005540](http://www.ncbi.nlm.nih.gov/entrez/viewer.fcgi?val=CY005540) | 1467 | A/duck/Hong Kong/319/1978(H2N2) |
| [JX081142](http://www.ncbi.nlm.nih.gov/entrez/viewer.fcgi?val=JX081142) | 1457 | A/emperor goose/Alaska/44297-260/2007(H2N2) |

**Table S3. Information from GenBank of the 72 Ebolaviruses**

| Accession  Number | Length  (nt) | Description |
| --- | --- | --- |
| KC545393.1 | 18939 | Bundibugyo ebolavirus isolate EboBund-112 2012, complete genome |
| KC545394.1 | 18939 | Bundibugyo ebolavirus isolate EboBund-120 2012, complete genome |
| KC545395.1 | 18939 | Bundibugyo ebolavirus isolate EboBund-122 2012, complete genome |
| KC545396.1 | 18939 | Bundibugyo ebolavirus isolate EboBund-14 2012, complete genome |
| FJ217161.1 | 18940 | Bundibugyo ebolavirus, complete genome |
| NC_014373.1 | 18940 | Bundibugyo ebolavirus isolate Bundibugyo virus |
| AF086833.2 | 18959 | Ebola virus - Mayinga, Zaire, 1976, complete genome |
| AF499101.1 | 18960 | Zaire Ebola virus strain Mayinga, complete genome |
| AY142960.1 | 18959 | Zaire Ebola virus strain Mayinga subtype Zaire, complete genome |
| NC_002549.1 | 18959 | Zaire ebolavirus isolate Ebola virus |
| AF272001.1 | 18959 | Zaire Ebola virus strain Mayinga, complete genome |
| KC242801.1 | 18959 | Zaire ebolavirus isolate EBOV |
| KC242791.1 | 18959 | Zaire ebolavirus isolate EBOV |
| KC242792.1 | 18959 | Zaire ebolavirus isolate EBOV |
| AY354458.1 | 18961 | Zaire ebolavirus strain Zaire 1995, complete genome |
| KC242796.1 | 18959 | Zaire ebolavirus isolate EBOV |
| KC242799.1 | 18959 | Zaire ebolavirus isolate EBOV |
| KC242793.1 | 18958 | Zaire ebolavirus isolate EBOV |
| KC242794.1 | 18959 | Zaire ebolavirus isolate EBOV |
| KC242795.1 | 18958 | Zaire ebolavirus isolate EBOV |
| KC242797.1 | 18958 | Zaire ebolavirus isolate EBOV |
| KC242798.1 | 18959 | Zaire ebolavirus isolate EBOV |
| KC242800.1 | 18958 | Zaire ebolavirus isolate EBOV |
| KC242784.1 | 18958 | Zaire ebolavirus isolate EBOV |
| KC242785.1 | 18958 | Zaire ebolavirus isolate EBOV |
| KC242786.1 | 18958 | Zaire ebolavirus isolate EBOV |
| KC242787.1 | 18958 | Zaire ebolavirus isolate EBOV |
| KC242788.1 | 18958 | Zaire ebolavirus isolate EBOV |
| KC242789.1 | 18958 | Zaire ebolavirus isolate EBOV |
| KC242790.1 | 18958 | Zaire ebolavirus isolate EBOV |
| KJ660346.2 | 18959 | Zaire ebolavirus isolate H.sapiens-wt |
| KJ660347.2 | 18959 | Zaire ebolavirus isolate H.sapiens-wt |
| KJ660348.2 | 18959 | Zaire ebolavirus isolate H.sapiens-wt |
| JQ352763.1 | 18944 | Zaire ebolavirus strain Kikwit, complete genome |
| HQ613403.1 | 18807 | Zaire ebolavirus isolate M-M, partial genome |
| HQ613402.1 | 18774 | Zaire ebolavirus isolate 034-KS, partial genome |
| AF522874.1 | 18891 | Reston Ebola virus strain Pennsylvania, complete genome |
| JX477166.1 | 18891 | Reston ebolavirus isolate RESTV |
| FJ621583.1 | 18887 | Reston ebolavirus - Reston strain Reston08-A, complete genome |
| FJ621584.1 | 18836 | Reston ebolavirus - Reston strain Reston08-C, complete genome |
| FJ621585.1 | 18796 | Reston ebolavirus - Reston strain Reston08-E, complete genome |
| JX477165.1 | 18887 | Reston ebolavirus isolate RESTV |
| AB050936.1 | 18890 | Reston ebolavirus - Reston genomic RNA, complete genome |
| AY769362.1 | 18895 | Reston ebolavirus strain Pennsylvania, complete genome |
| NC_004161.1 | 18891 | Reston ebolavirus isolate Reston virus |
| KC242783.2 | 18875 | Sudan ebolavirus isolate SUDV |
| AY729654.1 | 18875 | Sudan ebolavirus strain Gulu, complete genome |
| EU338380.1 | 18875 | Sudan ebolavirus isolate EBOV-S-2004 from Sudan, complete genome |
| FJ968794.1 | 18875 | Sudan ebolavirus strain Boniface, complete genome |
| KC545389.1 | 18874 | Sudan ebolavirus isolate EboSud-602 2012, complete genome |
| KC545390.1 | 18874 | Sudan ebolavirus isolate EboSud-603 2012, complete genome |
| KC545391.1 | 18874 | Sudan ebolavirus isolate EboSud-609 2012, complete genome |
| KC545392.1 | 18874 | Sudan ebolavirus isolate EboSud-682 2012, complete genome |
| KC589025.1 | 18875 | Sudan ebolavirus isolate EboSud-639, complete genome |
| JN638998.1 | 18875 | Sudan ebolavirus - Nakisamata, complete genome |
| NC_006432.1 | 18875 | Sudan ebolavirus isolate Sudan virus |
| FJ217162.1 | 18935 | Cote dIvoire ebolavirus, complete genome |
| KM034562.1 | 18957 | Zaire ebolavirus isolate Ebola virus |
| KM233050.1 | 18956 | Zaire ebolavirus isolate Ebola virus |
| KM233096.1 | 18953 | Zaire ebolavirus isolate Ebola virus |
| KM233109.1 | 18958 | Zaire ebolavirus isolate Ebola virus |
| KM233113.1 | 18958 | Zaire ebolavirus isolate Ebola virus |
| JN408064.1 | 19113 | Lake Victoria marburgvirus - Leiden, complete genome |
| KC545388.1 | 19114 | Marburg marburgvirus isolate Mbg-423-2012, complete genome |
| JX458852.1 | 19114 | Marburg marburgvirus isolate MARV |
| JX458851.1 | 19114 | Marburg marburgvirus isolate MARV |
| NC_001608.3 | 19111 | Marburg marburgvirus isolate Marburg virus |
| DQ447649.1 | 19114 | Lake Victoria marburgvirus - Ravn, complete genome |
| NC_016144.1 | 18927 | Lloviu cuevavirus isolate Lloviu virus |
| KM233111.1 | 18749 | Zaire ebolavirus isolate Ebola virus |
| KM233083.1 | 18773 | Zaire ebolavirus isolate Ebola virus |
| KM034552.1 | 18769 | Zaire ebolavirus isolate Ebola virus |

**Table S4. Information from GenBank of the 351 viruses**

| Accession  Number | Length  (nt) | Description |
| --- | --- | --- |
| NC_018872.2 | 9512 | Pokeweed mosaic virus |
| NC_022961.1 | 8424 | Garlic virus D |
| NC_001786.1 | 11488 | Barmah Forest virus |
| NC_003900.1 | 11824 | Aura virus |
| NC_022072.1 | 18643 | Blackberry vein banding associated virus |
| NC_002306.3 | 29355 | Feline infectious peritonitis virus |
| NC_003436.1 | 28033 | Porcine epidemic diarrhea virus |
| NC_005147.1 | 30738 | Human coronavirus OC43 |
| NC_007447.1 | 28475 | Breda virus |
| NC_010800.1 | 27657 | Turkey coronavirus |
| NC_018871.1 | 28494 | Rousettus bat coronavirus HKU10 |
| NC_001639.1 | 14104 | Lactate dehydrogenase-elevating virus |
| NC_015668.1 | 20128 | Cavally virus |
| AC_000192.1 | 31526 | Murine hepatitis virus strain JHM |
| NC_021786.1 | 9499 | Habenaria mosaic virus |
| NC_023175.1 | 10346 | Zucchini tigre mosaic virus |
| NC_023760.1 | 28941 | Mink coronavirus strain WD1127 |
| NC_002058.3 | 7440 | Enterovirus C |
| NC_023812.1 | 11624 | Madariaga virus |
| NC_001490.1 | 7212 | Human rhinovirus B14 |
| NC_003988.1 | 7374 | Enterovirus H |
| NC_001472.1 | 7389 | Enterovirus B |
| NC_001617.1 | 7152 | Rhinovirus A |
| NC_001612.1 | 7413 | Enterovirus A |
| NC_004441.1 | 7388 | Enterovirus G |
| NC_009996.1 | 7099 | Rhinovirus C |
| NC_001430.1 | 7390 | Enterovirus D |
| NC_021220.1 | 7397 | Enterovirus F |
| NC_008714.1 | 7390 | Possum enterovirus W1 |
| NC_022787.1 | 28301 | Porcine torovirus |
| NC_023892.1 | 8659 | Gaillardia latent virus |
| NC_022643.1 | 30175 | Betacoronavirus Erinaceus/VMC/DEU/2012 |
| NC_001265.2 | 4003 | Carnation mottle virus |
| NC_024377.1 | 9525 | Simian pegivirus |
| NC_024448.1 | 15362 | Mint-like virus |
| NC_001859.1 | 7414 | Enterovirus E |
| NC_019843.3 | 30119 | Middle East respiratory syndrome coronavirus |
| NC_024906.1 | 17656 | Rose leaf rosette-associated virus |
| NC_022103.1 | 28035 | Bat coronavirus CDPHE15/USA/2006 |
| NC_025217.1 | 31491 | Bat Hp-betacoronavirus/Zhejiang2013 |
| NC_023422.1 | 8305 | Caprine kobuvirus |
| NC_023628.1 | 9687 | Narcissus late season yellows virus |
| NC_025435.1 | 5986 | Strawberry polerovirus 1 |
| NC_025789.1 | 8336 | Garlic virus B |
| NC_025837.1 | 5820 | Sauropus yellowing virus |
| NC_003092.2 | 15717 | Simian hemorrhagic fever virus |
| NC_017083.1 | 31100 | Rabbit coronavirus HKU14 |
| NC_009087.2 | 8855 | Chrysanthemum virus B |
| NC_016990.1 | 25437 | Porcine coronavirus HKU15 |
| NC_016991.1 | 26041 | White-eye coronavirus HKU16 |
| NC_016993.1 | 26689 | Magpie-robin coronavirus HKU18 |
| NC_016994.1 | 26077 | Night-heron coronavirus HKU19 |
| NC_016995.1 | 26227 | Wigeon coronavirus HKU20 |
| NC_016996.1 | 26223 | Common-moorhen coronavirus HKU21 |
| NC_001837.1 | 9550 | Pegivirus A |
| NC_001441.1 | 6955 | Narcissus mosaic virus |
| NC_002160.2 | 5695 | Barley yellow dwarf virus-PAS |
| NC_002551.1 | 8284 | Vesicular exanthema of swine virus |
| NC_001642.1 | 6366 | Bamboo mosaic virus |
| NC_003375.1 | 8660 | Garlic virus A |
| NC_003376.1 | 8405 | Garlic virus C |
| NC_003487.1 | 3762 | Tobacco necrosis virus D |
| NC_003535.1 | 4029 | Cowpea mottle virus |
| NC_003632.1 | 7059 | Potato aucuba mosaic virus |
| NC_003678.1 | 12602 | Pestivirus Giraffe-1 |
| NC_003680.1 | 5273 | Barley yellow dwarf virus-MAV |
| NC_003795.1 | 8832 | Shallot virus X |
| NC_003908.1 | 11484 | Western equine encephalitis virus |
| NC_004012.1 | 8451 | Garlic virus E |
| NC_001481.2 | 7683 | Feline calicivirus |
| NC_004724.1 | 16527 | Grapevine rootstock stem lesion associated virus |
| NC_004752.1 | 9608 | Yam mosaic virus |
| NC_005064.1 | 11375 | Kamiti River virus |
| NC_005136.1 | 9346 | Oat necrotic mottle virus |
| NC_002645.1 | 27317 | Human coronavirus 229E |
| NC_005286.1 | 3923 | Pelargonium flower break virus |
| NC_005304.1 | 9591 | Beet mosaic virus |
| NC_004451.1 | 8126 | Simian sapelovirus 1 |
| NC_004421.1 | 8374 | Bovine kobuvirus |
| NC_004452.3 | 3644 | Beet black scorch virus |
| NC_005831.2 | 27553 | Human coronavirus NL63 |
| NC_002598.1 | 4326 | Panicum mosaic virus |
| NC_001780.1 | 3879 | Saguaro cactus virus |
| NC_001800.1 | 8106 | Garlic virus X |
| NC_003398.1 | 9596 | Sugarcane mosaic virus |
| NC_002815.2 | 6614 | Cactus virus X |
| NC_003794.1 | 5966 | Strawberry mild yellow edge virus |
| NC_005138.1 | 8394 | Lily symptomless virus |
| NC_004666.1 | 5685 | Barley yellow dwarf virus-GAV |
| NC_006550.1 | 9104 | Sweet potato chlorotic fleck virus |
| NC_006553.1 | 8289 | Avian sapelovirus |
| NC_006262.1 | 10035 | Watermelon mosaic virus |
| NC_006551.1 | 11066 | Usutu virus |
| NC_005343.1 | 8741 | Poplar mosaic virus |
| NC_006265.1 | 5723 | Carrot red leaf virus |
| NC_005065.1 | 15045 | Little cherry virus 2 |
| NC_005903.1 | 9540 | Agropyron mosaic virus |
| NC_005904.1 | 9463 | Hordeum mosaic virus |
| NC_002615.1 | 7442 | European brown hare syndrome virus |
| NC_002032.1 | 12255 | Bovine viral diarrhea virus 2 |
| NC_002187.1 | 4014 | Japanese iris necrotic ring virus |
| NC_004035.1 | 9624 | Sorghum mosaic virus |
| NC_006577.2 | 29926 | Human coronavirus HKU1 |
| NC_006943.1 | 6185 | Hydrangea ringspot virus |
| NC_006941.1 | 9751 | Cucumber vein yellowing virus |
| NC_003397.1 | 9992 | Bean common mosaic virus |
| NC_004011.1 | 10142 | Leek yellow stripe virus |
| NC_003930.1 | 11919 | Salmon pancreas disease virus |
| NC_003399.1 | 9324 | Scallion mosaic virus |
| NC_004750.1 | 5677 | Barley yellow dwarf virus-PAV |
| NC_003982.1 | 7734 | Equine rhinitis A virus |
| NC_003608.1 | 3911 | Hibiscus chlorotic ringspot virus |
| NC_006944.1 | 15450 | Mint virus 1 |
| NC_005778.1 | 9711 | Chilli veinal mottle virus |
| NC_003215.1 | 11442 | Semliki forest virus |
| NC_002031.1 | 10862 | Yellow fever virus |
| NC_001785.1 | 10326 | Papaya ringspot virus |
| NC_001616.1 | 9704 | Potato virus Y |
| NC_001671.1 | 9924 | Pea seed-borne mosaic virus |
| NC_001445.1 | 9741 | Plum pox virus |
| NC_001449.1 | 11444 | Venezuelan equine encephalitis virus |
| NC_001451.1 | 27608 | Infectious bronchitis virus |
| NC_003687.1 | 10839 | Powassan virus |
| NC_001461.1 | 12573 | Bovine viral diarrhea virus 1 |
| NC_001477.1 | 10735 | Dengue virus 1 |
| NC_001479.1 | 7835 | Encephalomyocarditis virus |
| NC_001437.1 | 10976 | Japanese encephalitis virus |
| NC_001512.1 | 11835 | O'nyong-nyong virus |
| NC_001517.1 | 9640 | Pepper mottle virus |
| NC_001543.1 | 7437 | Rabbit hemorrhagic disease virus |
| NC_001544.1 | 11657 | Ross River virus |
| NC_001547.1 | 11703 | Sindbis virus |
| NC_001361.2 | 8533 | Potato virus M |
| NC_001555.1 | 9494 | Tobacco etch virus |
| NC_001564.1 | 10695 | Cell fusing agent virus |
| NC_001598.1 | 15480 | Beet yellows virus |
| NC_003499.1 | 8512 | Blueberry scorch virus |
| NC_001632.1 | 12226 | Rice tungro spherical virus |
| NC_004047.1 | 9612 | Bean common mosaic necrosis virus |
| NC_001661.1 | 19296 | Citrus tristeza virus |
| NC_001672.1 | 11141 | Tick-borne encephalitis virus |
| NC_003501.1 | 9672 | Brome streak mosaic virus |
| NC_003492.1 | 9532 | Bean yellow mosaic virus |
| NC_003797.1 | 10818 | Sweet potato mild mottle virus |
| NC_003605.1 | 10080 | Lettuce mosaic virus |
| NC_001809.1 | 10871 | Louping ill virus |
| NC_001814.1 | 9535 | Ryegrass mosaic virus |
| NC_003626.1 | 11832 | Maize chlorotic dwarf virus |
| NC_001836.1 | 16934 | Little cherry virus 1 |
| NC_001841.1 | 10820 | Sweet potato feathery mottle virus |
| NC_001768.1 | 9475 | Tobacco vein mottling virus |
| NC_003606.1 | 9779 | Johnsongrass mosaic virus |
| NC_001846.1 | 31357 | Murine hepatitis virus strain A59 |
| NC_002600.1 | 9709 | Peanut mottle virus |
| NC_003536.1 | 9584 | Clover yellow vein virus |
| NC_001886.1 | 9384 | Wheat streak mosaic virus |
| NC_003377.1 | 9515 | Maize dwarf mosaic virus |
| NC_001897.1 | 7348 | Human parechovirus |
| NC_000947.1 | 9760 | Japanese yam mosaic virus |
| NC_003675.1 | 10140 | Rio Bravo virus |
| NC_003676.1 | 10116 | Apoi virus |
| NC_003690.1 | 10943 | Langat virus |
| NC_002552.1 | 8612 | Hop latent virus |
| NC_004039.1 | 9585 | Potato virus A |
| NC_002634.1 | 9588 | Soybean mosaic virus |
| NC_004010.1 | 9848 | Potato virus V |
| NC_002795.1 | 8657 | Aconitum latent virus |
| NC_002532.2 | 12704 | Equine arteritis virus |
| NC_003045.1 | 31028 | Bovine coronavirus |
| NC_004355.1 | 10685 | Alkhumra hemorrhagic fever virus |
| NC_003537.1 | 10038 | Dasheen mosaic virus |
| NC_003224.1 | 9591 | Zucchini yellow mosaic virus |
| NC_003417.1 | 11411 | Mayaro virus |
| NC_003635.1 | 10600 | Modoc virus |
| NC_004013.1 | 9465 | Cowpea aphid-borne mosaic virus |
| NC_003433.1 | 11900 | Sleeping disease virus |
| NC_003996.1 | 10053 | Tamana bat virus |
| NC_003742.1 | 9663 | Cocksfoot streak virus |
| NC_003987.1 | 7491 | Porcine sapelovirus 1 |
| NC_003976.2 | 7590 | Ljungan virus |
| NC_004119.1 | 10690 | Montana myotis leukoencephalitis virus |
| NC_004426.1 | 9878 | Wild potato mosaic virus |
| NC_005028.1 | 10155 | Papaya leaf distortion mosaic virus |
| NC_004573.1 | 9899 | Peru tomato mosaic virus |
| NC_005029.1 | 10538 | Onion yellow dwarf virus |
| NC_002509.2 | 9835 | Turnip mosaic virus |
| NC_003557.1 | 8363 | Garlic latent virus |
| NC_003899.1 | 11675 | Eastern equine encephalitis virus |
| NC_000943.1 | 11014 | Murray Valley encephalitis virus |
| NC_004102.1 | 9646 | Hepatitis C virus |
| NC_001918.1 | 8251 | Aichi virus 1 |
| NC_007147.1 | 9611 | Pennisetum mosaic virus |
| NC_002657.1 | 12301 | Classical swine fever virus |
| NC_003679.1 | 12333 | Border disease virus |
| NC_001710.1 | 9392 | GB virus C |
| NC_007180.1 | 9723 | Thunberg fritillary virus |
| NC_005288.1 | 9644 | Lily mottle virus |
| NC_004718.3 | 29751 | SARS coronavirus |
| NC_001504.1 | 4266 | Melon necrotic spot virus |
| NC_001959.2 | 7654 | Norwalk virus |
| NC_007216.1 | 9695 | Wisteria vein mosaic virus |
| NC_007289.1 | 8478 | Potato virus S |
| NC_001475.2 | 10707 | Dengue virus 3 |
| NC_002640.1 | 10649 | Dengue virus 4 |
| NC_007433.1 | 10429 | Shallot yellow stripe virus |
| NC_007448.1 | 16494 | Grapevine leafroll-associated virus 2 |
| NC_017824.1 | 10057 | Tomato necrotic stunt virus |
| NC_017859.1 | 8601 | American hop latent virus |
| NC_007728.1 | 10046 | East Asian Passiflora virus |
| NC_007732.1 | 30480 | Porcine hemagglutinating encephalomyelitis virus |
| NC_007733.1 | 3964 | Angelonia flower break virus |
| NC_007913.1 | 9544 | Konjac mosaic virus |
| NC_008020.1 | 8739 | Daphne virus S |
| NC_017977.1 | 9908 | Vallota speciosa virus |
| NC_017967.1 | 9660 | Hippeastrum mosaic virus |
| NC_008028.1 | 9548 | Daphne mosaic virus |
| NC_016992.1 | 26083 | Sparrow coronavirus HKU17 |
| NC_008266.1 | 8539 | Narcissus common latent virus |
| NC_008292.1 | 8386 | Passiflora latent carlavirus |
| NC_008311.1 | 7382 | Murine norovirus 1 |
| NC_008315.1 | 30307 | Bat coronavirus (BtCoV/133/2005) |
| NC_008366.1 | 17039 | Strawberry chlorotic fleck-associated virus |
| NC_008393.1 | 9890 | Pepper severe mosaic virus |
| NC_008552.1 | 8281 | Narcissus symptomless virus |
| NC_008585.1 | 17481 | Raspberry leaf mottle virus |
| NC_008604.2 | 10837 | Culex flavivirus |
| NC_008824.1 | 9816 | Narcissus degeneration virus |
| NC_009019.1 | 30286 | Tylonycteris bat coronavirus HKU4 |
| NC_009020.1 | 30482 | Pipistrellus bat coronavirus HKU5 |
| NC_009021.1 | 29114 | Rousettus bat coronavirus HKU9 |
| NC_009383.1 | 8590 | Phlox virus S |
| NC_009448.2 | 8115 | Saffold virus |
| NC_001474.2 | 10723 | Dengue virus 2 |
| NC_009745.1 | 9711 | Banana bract mosaic virus |
| NC_009741.1 | 9804 | Basella rugose mosaic virus |
| NC_009742.1 | 9689 | Telosma mosaic virus |
| NC_009744.1 | 9659 | Wild tomato mosaic virus |
| NC_009759.1 | 8404 | Potato Virus P |
| NC_009764.1 | 8727 | Coleus vein necrosis virus |
| NC_009805.1 | 9636 | Wheat eqlid mosaic virus |
| NC_009824.1 | 9456 | Hepatitis C virus genotype 3 |
| NC_009994.1 | 9570 | Tobacco vein banding mosaic virus |
| NC_009995.1 | 9730 | Moroccan watermelon mosaic virus |
| NC_009991.1 | 9058 | Phlox Virus B |
| NC_009992.1 | 14214 | Plum bark necrosis stem pitting-associated virus |
| NC_009988.1 | 27165 | Rhinolophus bat coronavirus HKU2 |
| NC_010178.1 | 13071 | Pineapple mealybug wilt-associated virus 1 |
| NC_010305.1 | 8412 | Ligustrum necrotic ringspot virus |
| NC_010327.1 | 30992 | Equine coronavirus |
| NC_010354.1 | 7556 | Bovine rhinitis B virus |
| NC_010521.1 | 9836 | Squash vein yellowing virus |
| NC_010538.1 | 8550 | Hop mosaic virus |
| NC_010438.1 | 28773 | Miniopterus bat coronavirus HKU8 |
| NC_010437.1 | 28326 | Bat coronavirus 1A |
| NC_010436.1 | 28476 | Bat coronavirus 1B |
| NC_010646.1 | 31686 | Beluga Whale coronavirus SW1 |
| NC_010735.1 | 9742 | Verbena virus Y |
| NC_010736.1 | 9842 | Algerian watermelon mosaic virus |
| NC_010806.1 | 5808 | Rose spring dwarf-associated virus |
| NC_010954.1 | 9656 | Fritillary virus Y |
| NC_011525.1 | 7890 | Potato latent virus |
| NC_011540.1 | 8500 | Hippeastrum latent virus |
| NC_011541.1 | 9650 | Narcissus yellow stripe virus |
| NC_011560.1 | 9973 | Zantedeschia mild mosaic virus |
| NC_011702.1 | 13696 | Grapevine leafroll-associated virus 10 |
| NC_011829.1 | 8210 | Porcine kobuvirus swine/S-1-HUN/2007/Hungary |
| NC_011918.1 | 9792 | Pepper veinal mottle virus |
| NC_012038.1 | 8542 | Helleborus net necrosis virus |
| NC_012210.1 | 8604 | Red clover vein mosaic virus |
| NC_012561.1 | 11526 | Highlands J virus |
| NC_012798.1 | 6580 | Human cosavirus E |
| NC_012799.1 | 10282 | Triticum mosaic virus |
| NC_012801.1 | 7205 | Human cosavirus B |
| NC_012802.1 | 7215 | Human cosavirus D |
| NC_012869.1 | 8433 | Hydrangea chlorotic mottle virus |
| NC_012949.1 | 30953 | Bovine respiratory coronavirus bovine/US/OH-440-TC/1996 |
| NC_013006.1 | 8517 | Kalanchoe latent virus |
| NC_013261.1 | 9502 | Canna yellow streak virus |
| NC_013527.1 | 8662 | Butterbur mosaic virus |
| NC_013528.1 | 11381 | Fort Morgan virus |
| NC_014037.1 | 9782 | Sugarcane streak mosaic virus |
| NC_014038.1 | 9965 | Sunflower chlorotic mottle virus |
| NC_014064.1 | 9489 | Freesia mosaic virus |
| NC_014252.1 | 9750 | Panax virus Y |
| NC_014325.1 | 9741 | Bidens mottle virus |
| NC_014412.1 | 7641 | Oscivirus A1 |
| NC_014413.1 | 7678 | Oscivirus A2 |
| NC_014470.1 | 29276 | Bat coronavirus BM48-31/BGR/2008 |
| NC_014536.1 | 9870 | Brugmansia suaveolens mottle virus |
| NC_014545.1 | 5866 | Cotton leafroll dwarf virus |
| NC_014730.1 | 8127 | Cowpea mild mottle virus |
| NC_014742.1 | 10820 | Sweet potato virus C |
| NC_014898.1 | 10113 | Lupine mosaic virus |
| NC_014905.1 | 9917 | Apium virus Y |
| NC_014967.1 | 3956 | Honeysuckle ringspot virus |
| NC_015393.1 | 9999 | Celery mosaic virus |
| NC_015394.2 | 9682 | Hardenbergia mosaic virus |
| NC_015874.1 | 20192 | Nam Dinh virus |
| NC_016044.1 | 9595 | Chilli ringspot virus |
| NC_016080.1 | 8315 | Mirabilis jalapa mottle virus |
| NC_016081.1 | 13384 | Grapevine leafroll-associated virus 5 |
| NC_016416.1 | 13830 | Grapevine leafroll-associated virus 4 |
| NC_016417.1 | 13807 | Grapevine leafroll-associated virus 6 |
| NC_016436.1 | 16404 | Grapevine leafroll-associated virus 7 |
| NC_016440.1 | 8638 | Garlic common latent virus |
| NC_016441.1 | 9649 | Yam bean mosaic virus |
| NC_016509.1 | 18659 | Grapevine leafroll-associated virus 1 |
| NC_018571.1 | 5822 | Suakwa aphid-borne yellows virus |
| NC_001961.1 | 15428 | Porcine reproductive and respiratory syndrome virus |
| NC_019409.1 | 9445 | Ornithogalum mosaic virus |
| NC_019415.1 | 9842 | Blue squill virus A |
| NC_011108.1 | 4198 | Cocksfoot mild mosaic virus |
| NC_012936.1 | 31250 | Rat coronavirus Parker |
| NC_012948.1 | 30969 | Bovine respiratory coronavirus AH187 |
| NC_012950.1 | 30953 | Human enteric coronavirus strain 4408 |
| NC_013007.1 | 16354 | Carrot yellow leaf virus |
| NC_009657.1 | 28203 | Scotophilus bat coronavirus 512 |
| NC_018572.1 | 9847 | Caladenia virus A |
| NC_014791.1 | 9070 | Ugandan cassava brown streak virus |
| NC_012698.2 | 8995 | Cassava brown streak virus |
| NC_019412.1 | 9538 | Yam mild mosaic virus |
| NC_001747.1 | 5987 | Potato leafroll virus |
| NC_014790.2 | 9682 | Passion fruit woodiness virus |
| NC_003491.1 | 5722 | Beet mild yellowing virus |
| NC_002766.1 | 5776 | Beet chlorosis virus |
| NC_004756.1 | 5666 | Beet western yellows virus |
| NC_002198.2 | 5662 | Cereal yellow dwarf virus-RPS |
| NC_004751.1 | 5723 | Cereal yellow dwarf virus-RPV |
| NC_000874.1 | 5899 | Sugarcane yellow leaf virus |
| NC_003688.1 | 5669 | Cucurbit aphid-borne yellows virus |
| NC_008249.1 | 5900 | Chickpea chlorotic stunt virus |
| NC_010809.1 | 5674 | Melon aphid-borne yellows virus |
| NC_003743.1 | 5641 | Turnip yellows virus |
| NC_010732.1 | 5920 | Tobacco vein distorting virus |
| NC_016038.1 | 5666 | Brassica yellows virus |
| NC_012931.1 | 5673 | Wheat yellow dwarf virus-GPV |
| NC_018093.1 | 10798 | Sweet potato virus G |
| NC_021153.1 | 8879 | Rodent hepacivirus |
| NC_021154.1 | 11279 | Rodent pegivirus |
| NC_004004.1 | 8134 | Foot-and-mouth disease virus - type O |
| NC_012800.1 | 7632 | Cosavirus A |
| NC_021197.1 | 9868 | Donkey orchid virus A |
| NC_018175.1 | 8410 | Potato virus H |
| NC_015050.1 | 6244 | Pepper vein yellows virus |
| NC_021481.1 | 5736 | Barley yellow dwarf virus Ker-II |
| NC_021482.1 | 7537 | Sebokele virus 1 |
| NC_010810.1 | 7961 | Human TMEV-like cardiovirus |
| NC_001366.1 | 8101 | Theilovirus |
| NC_021484.1 | 5612 | Maize yellow dwarf virus-RMV |
| NC_004667.1 | 17919 | Grapevine leafroll-associated virus 3 |
| NC_021705.2 | 4195 | Thin paspalum asymptomatic virus |

**Table S5. Information from Accession Number of mitochondrial genomes from 31 mammals**

| **Accession Number** | **Official Name** | **Order** | **Full Name** |
| --- | --- | --- | --- |
| V00662 | Homo sapiens | Primates | Primates Homo sapiens |
| D38116 | Pan paniscus | Primates | Primates Pan paniscus |
| D38113 | Pan troglodytes | Primates | Primates Pan troglodytes |
| X99256 | Nomascus leucogenys | Primates | Primates Nomascus leucogenys |
| Y18001 | Papio anubis | Primates | Primates Papio anubis |
| AY863426 | Cercopithecus aethiops | Primates | Primates Cercopithecus aethiops |
| NC_002764 | Macaca sylvanus | Primates | Primates Macaca sylvanus |
| D38115 | Pongo pygmaeus | Primates | Primates Pongo pygmaeus |
| NC_002083 | Pongo abelii | Primates | Primates Pongo abelii |
| D38114 | Gorilla gorilla | Primates | Primates Gorilla gorilla |
| U20753 | Felis catus | [Carnivora](https://en.wikipedia.org/wiki/Carnivora) | Carnivora Felis catus |
| U96639 | Canis familiaris | [Carnivora](https://en.wikipedia.org/wiki/Carnivora) | Carnivora Canis familiaris |
| AJ002189 | Sus scrofa | Cetartiodactyla | Cetartiodactyla Sus scrofa |
| AF010406 | Ovis aries | Cetartiodactyla | Cetartiodactyla Ovis aries |
| AF533441 | Capra hircus | Cetartiodactyla | Cetartiodactyla Capra hircus |
| V00654 | Bos taurus | Cetartiodactyla | Cetartiodactyla Bos taurus |
| AY488491 | Bubalus bubalis | Cetartiodactyla | Cetartiodactyla Bubalus bubalis |
| EU442884 | Canis lupus chanco | Carnivora | Carnivora Canis lupus chanco |
| EF551003 | Amus/Panthera tigris | [Carnivora](https://en.wikipedia.org/wiki/Carnivora) | Carnivora Amus/Panthera tigris |
| EF551002 | Panthera pardus | [Carnivora](https://en.wikipedia.org/wiki/Carnivora) | Carnivora Panthera pardus |
| X97336 | Rhinoceros unicornis | Perissodactyla | Perissodactyla Rhinoceros unicornis |
| Y07726 | Ceratotherium simum | Perissodactyla | Perissodactyla Ceratotherium simum |
| DQ402478 | Ursus thibetanus mupinensis | [Carnivora](https://en.wikipedia.org/wiki/Carnivora) | Carnivora Ursus thibetanus mupinensis |
| AF303110 | Ursus arctos | [Carnivora](https://en.wikipedia.org/wiki/Carnivora) | Carnivora Ursus arctos |
| AF303111 | Ursus maritimus | [Carnivora](https://en.wikipedia.org/wiki/Carnivora) | Carnivora Ursus maritimus |
| EF212882 | Ailuropoda melanoleuca | [Carnivora](https://en.wikipedia.org/wiki/Carnivora) | Carnivora Ailuropoda melanoleuca |
| EF535828 | Ochotona curzoniae | Lagomorpha | Lagomorpha Ochotona curzoniae |
| X88898 | Erinaceus europaeus | Eulipotyphla | Eulipotyphla Erinaceus europaeus |
| AJ001562 | Myoxus glis | Rodentia | Rodentia Myoxus glis |
| AJ238588 | Sciurus vulgaris | Rodentia | Rodentia Sciurus vulgaris |
| X72204 | Balaenoptera musculus | Cetartiodactyla | Cetartiodactyla Balaenoptera musculus |

**Table S6. Information for simulation datasets**

| **Sequence Name** | **Description** |
| --- | --- |
| A_original | AAGAGTGTATACGTGCAAGTATTGACGACCACTACTTTAATGGTGATGCTACGGAAAGGATACTCCCTGAATGCTGGAGTGCGTACTAGCTGTCGGAAATCCCCGCGACAACACTTCGCCCCAACAGCACGTTAACATCGTCGCCAAGAACCCACATAGGAGCGTTCAAACACCGGTCTACGTAATGTTTCCCCATCACTGCCCTGCTGAAAGACAGTAATGCAGCGATTAGTAAAAGGCAGCGATACCCCTAATTTACCCTACTAAAGCAGTTACCGGACATCCAGCCGTTTATCACATTGAGATGAAAGTTTGCAAGCAGGCCTTGGGGACGGATCGAGACGAGTATTCGGACAGGGAGCGGGCCGTTGGGAAGGGAAGCCACATGAATTTTACGCTCCGGGAAATGAAGCATCAGATGGGTTGAAAGTGGAGCTTCTAAAACCCGACCGGGATAGGCGGTAGTCAAGTGTATTTACATATAAAGAACCGAGAAATAGAAGGTGTGCCGCGTGATCGGTGATTTCGGGTTATTTAGGACCGAATCCAGCTAAACGGCAGGAAGCCCTCTGTCTATTCACATCACGAGGCCCGTTCCAGGTAGTATTCTGACGACGACGCGGACTTGAATTGGGAGCGCCACCCCTACTTATCTTTAAATTATTACTCGGGCCCATATCCAGTCAGGAAGGAGTCGTGGTATTGCGATAGATCCACTTCCGGTCCTATTTGCGGCGAACAGCAATTCAATATGATGATAGATGAATTCGTCCCTAGCCTTCCCGGTCATCCAACTACCATACAGACTATCGGACCAGTATCGGTTCCTCCCCCACTAAAGGTGGGCCAGGTGGACAGGGCAGATAAAGCCCGCCTGTCGGGTCTCAGGGGTTCTAAGAACCAACCAATATTCATTAGATTATAAGTGTCCGACTCGCTCTACCTAAAGAGTGGCGCGTGTCACTTTAAGGATCAGACGGTCGAAGTGATCTGAGTAGAC |
| A1 | Mutation positions: 797, 856 |
| A2 | Mutation positions: 95, 903 |
| A3 | Mutation positions: 65, 96, 106, 153, 396 |
| A4 | Mutation positions: 127, 632, 815, 906, 913 |
| A5 | Mutation positions: 25, 87, 195, 208, 252, 468, 548, 616, 738, 866 |
| A6 | Mutation positions: 25, 93, 149, 187, 201, 310, 332, 681, 688, 735 |
| B_original | AAGAGTGTAGACGTGCACTTATTGACGACCACTACTTTAATGCTGATGCTACGGACAGGTTGCTCCCTGAGTGCTCGAGTGAGTACTAGCAGTCGGAAATCCCGGCCACAGCACTTCGCCCCCACAGCCCCTATACATCGTCGACAAGAACCCACATAGGACCCTTAAAACACCGGTCTACGTAATGTTTCCCCATCACTGCCCTCCTGAAACACCGTAATACAGCGATTGGTTAACGGCAGCGGTACCCCTAATTTACCTTACGAAACCAATTACCGGACATCCAGCCGTTTAACACAATGAGATGCAAGTTTGCAAGCAGGTCTTGCGGACGTATCGAGACGAGTATTCGGACAGAGAGGGGGACGTTGGGAACGGAAGCAACCTGCATTTTGCTCCCCGGGAAATGGAGCATTAGATCGGTTGAAAGTGGAGCTTCTGAAACTCGACCGGGAGAGTCTGTGGTCAAGTGTATTTACATATATACAACCCAAAGACAGAAGGCGTGGCGCGTGATCGGTGATTTCGGGTTATTTAGGACCGAATCAAACTAAACGGCAGGAAGCCCTCTGTTTATTCACATCACAAGCCCCGTTCCAGGTAGCTTTCTGACGAAGACGCTGATTTCTATTGGGAGCGGCACCCCTACTTAGCTTTAAAGTATTAGTCAGGCCAATATCCAGTCAGGCACGAGTTGTGGTATTACGATAGATCCACTTCCGGTAATCTTGCCAGCAAACAGCCAGTTAATCTGATGACAGATGAATTGTTCCCTTGCCTTCCCGGTCATCCAAATACCATACAGAATATCGGCGCAGTATCCGTTCCTCCCCCACTAAAGGTGGGCCAGGTCGACGAGGCAAGAGAAGCCCGCCCCTCCGGTCTCAGGGGTTCTACGAACCAACCGAAATTCATTACACTATAAGTGCCCGACTCGCTCTACCTAAAGAGTGGCGCGTCTCACTTTAAGAATCAGACCGTCGAGGTGATAGGAGTAGCC |
| B1 | Mutation positions: 98, 470 |
| B2 | Mutation positions: 70, 913 |
| B3 | Mutation positions: 47, 74, 500, 694, 913 |
| B4 | Mutation positions: 300, 591, 628, 733, 883 |
| B5 | Mutation positions: 79, 96, 108, 308, 414, 418, 559, 564, 795, 912 |
| B6 | Mutation positions: 143, 227, 259, 344, 784, 803, 830, 862, 881, 894 |
| B7 | 10 bp Deletion from positions 51:60 in B_original |
| B8 | 10 bp Deletion from positions 601:610 in B_original |
| B9 | 20 bp Insertion at position 51 in B_original (‘taaaccgtcgacgatttatg’) |
| B10 | 20 bp Insertion at position 601 in B_original (‘cactggtcgcatagtcaacg’) |
| B11 | 50 bp Transposition from position 1 to 50 in B_original |
| B12 | 100 bp Transposition from position 601 to 700 in B_original |

# Supplementary Figures


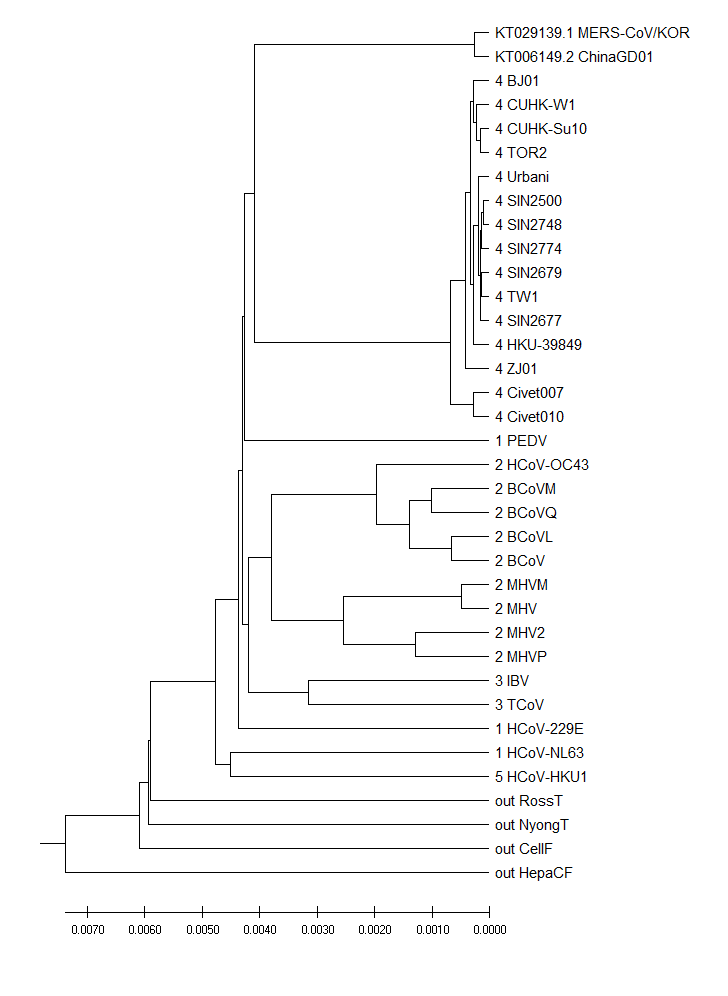
**Supplementary Figure S1.** The phylogenetic UPGMA tree using FFP (k-mer) method when $k=8$ on Coronaviruses dataset


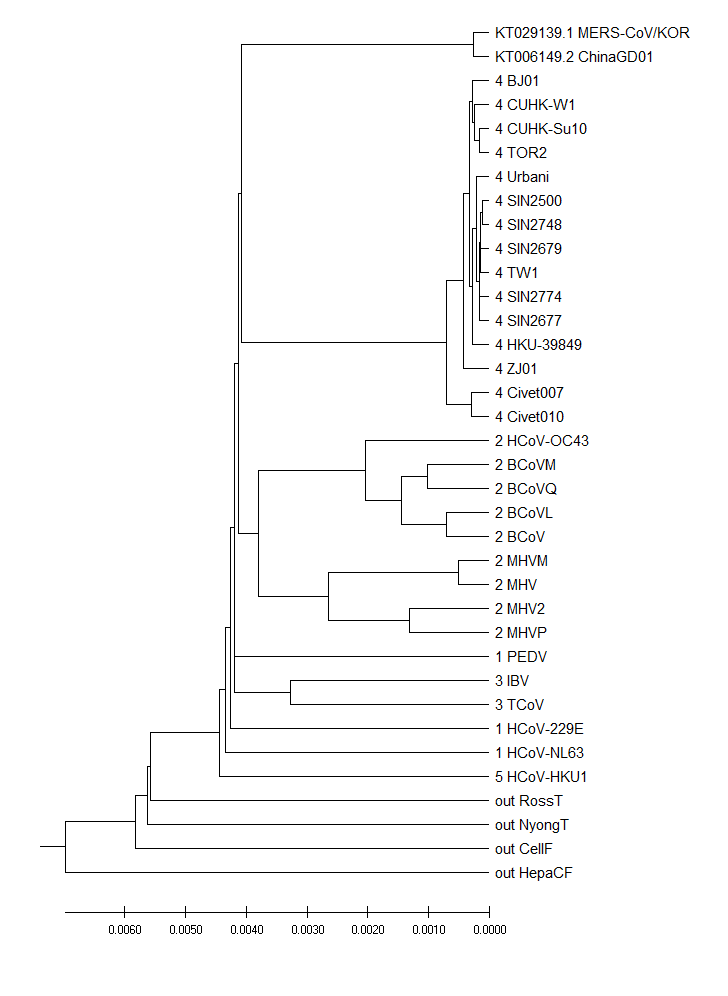


**Supplementary Figure S2.** The phylogenetic UPGMA tree using FFP (k-mer) method when $k=9$ on Coronaviruses dataset
